# What can we learn from interventions that aim to increase policy-makers’ capacity to use research? A realist scoping review

**DOI:** 10.1186/s12961-018-0277-1

**Published:** 2018-04-10

**Authors:** Abby Haynes, Samantha J. Rowbotham, Sally Redman, Sue Brennan, Anna Williamson, Gabriel Moore

**Affiliations:** 1 Sax Institute, 235 Jones Street, Ultimo, NSW 2007 Australia; 2 Sydney School of Public Health, Edward Ford Building (A27), University of Sydney, Camperdown, NSW 2006 Australia; 3 Menzies Centre for Health Policy, University of Sydney, Sydney, Australia; 4 The Australian Prevention Partnership Centre, Ultimo, NSW 2007 Australia; 5 Australasian Cochrane Centre, School of Public Health and Preventive Medicine, Monash University, Clayton, VIC 3800 Australia

**Keywords:** Realist review, Scoping review, Research utilisation, Knowledge mobilisation, Health policy, Capacity-building, Interventions

## Abstract

**Background:**

Health policy-making can benefit from more effective use of research. In many policy settings there is scope to increase capacity for using research individually and organisationally, but little is known about what strategies work best in which circumstances. This review addresses the question: What causal mechanisms can best explain the observed outcomes of interventions that aim to increase policy-makers’ capacity to use research in their work?

**Methods:**

Articles were identified from three available reviews and two databases (PAIS and WoS; 1999–2016). Using a realist approach, articles were reviewed for information about contexts, outcomes (including process effects) and possible causal mechanisms. Strategy + Context + Mechanism = Outcomes (SCMO) configurations were developed, drawing on theory and findings from other studies to develop tentative hypotheses that might be applicable across a range of intervention sites.

**Results:**

We found 22 studies that spanned 18 countries. There were two dominant design strategies (needs-based tailoring and multi-component design) and 18 intervention strategies targeting four domains of capacity, namely access to research, skills improvement, systems improvement and interaction. Many potential mechanisms were identified as well as some enduring contextual characteristics that all interventions should consider. The evidence was variable, but the SCMO analysis suggested that tailored interactive workshops supported by goal-focused mentoring, and genuine collaboration, seem particularly promising. Systems supports and platforms for cross-sector collaboration are likely to play crucial roles. Gaps in the literature are discussed.

**Conclusion:**

This exploratory review tentatively posits causal mechanisms that might explain how intervention strategies work in different contexts to build capacity for using research in policy-making.

**Electronic supplementary material:**

The online version of this article (10.1186/s12961-018-0277-1) contains supplementary material, which is available to authorized users.

## Background

There is widespread agreement that the use of research in policy-making could be improved, with potentially enormous social gains [1, 2]. There are disputes about the extent to which research can inform policy decision-making, and about what forms of research knowledge should be most valued and how they should be applied. However, these are underpinned by a shared belief that the effective use of relevant and robust research within policy processes is a good thing [3–6]; specifically, that research-informed policies can help prevent harm, maximise resources, tackle the serious challenges facing contemporary healthcare, and otherwise contribute to improved health outcomes [7–10]. Ensuring that health policy-makers have the capacity to access, generate and use research in decision-making is a priority.

Despite a rapidly growing body of literature about the use of research in policy-making, we have a limited understanding of how best to help policy-makers use research in their day-to-day work, partly because most of the literature is either descriptive or theoretical. Descriptive studies often struggle to identify findings that are transferable to other settings, which can limit their utility for informing intervention design [11]. Theoretical studies have produced many models, concepts and frameworks, but these are often hard to operationalise [12]. For example, Field et al. [13] found that one such framework, while frequently cited, was used with varying levels of completeness and seldom guided the actual design, delivery or evaluation of intervention activities. The authors conclude that prospective, primary research is needed to establish the real value of theoretical models and tools. Yet, testing specific strategies to increase or otherwise improve the use of research in policy processes is relatively underdeveloped [14, 15]. Consequently, we have a plethora of ideas about what may or may not support research-informed policy-making, but little robust empirical knowledge about what strategies are effective in which contexts.

This paper brings together information about interventions designed to build capacity for using research in policy processes and explores possible transferable lessons for future interventions.

### Using research in policy-making

The concept of research-informed policy has emerged from multiple disciplines with different paradigmatic influences, leading to debates about what we should expect of policy-making and the role of research within it [4, 16–18]. In summary, many reject what they see as inaccurate assumptions about the extent to which policy-making is a linear technical–rational process in which ‘objective’ academic research can be used instrumentally (i.e. to direct policy decision-making) [5, 16]. This argument is premised on policy being a rhetorical arena [19] where, often, facts are uncertain, values are contested, stakes high and decisions urgent [20]. Such views challenge the expectation that improved access to research, or greater capacity to use it, will result in increased use [21]. Indeed, several studies show that individual attributes and contextual factors frequently prevent the use of apparently helpful evidence (e.g. [22–25]). Some go further, questioning the assumption that there is a policy-research ‘gap’ that needs ‘bridging’, or that more use of research in policy-making will necessarily produce better health outcomes [26].

Counter arguments highlight the enormous number of policy-makers who are actively and effectively engaged (and often qualified) in using research, the success of strategies for improving research use such as policy dialogues, rapid review programs and partnership approaches [27–33], and the many cases where research has demonstrably influenced policy-making with positive results [2, 33–35]. From this perspective, research is only one source of evidence amongst many, but it is a critical source that has the potential to guide agendas, maximise the functioning of programs, support governments and service providers to act in the public interest, and hold them accountable when they get it wrong [2, 6, 36, 37]. As Lomas puts it, “*…the goal here is not for the imperial forces of research to vanquish all other inputs. Rather it is to have the role of research optimized in the context of other perfectly reasonable considerations…*” ([38], p. xiii).

### Building capacity to use research in policy-making

Capacity-building is conceptualised as a suite of strategies that seek to “*increase the self-sustaining ability of people to recognize, analyse and solve their problems by more effectively controlling and using their own and external resources*” ([39], p. 100). Thus, effective capacity-building interventions facilitate not merely technical skills development, but increased agency and agility [40].

Capacity is a multi-dimensional concept spanning different levels – individual, interpersonal, organisational and environmental [41, 42] – each of which is likely to require quite different intervention strategies and evaluation methods [43]. Capacity-building in policy agencies uses “*a variety of strategies that have to do with increasing the efficiency, effectiveness and responsiveness of government*” ([40], p. 212). In this context, performance and accountability are essential [4, 42, 44]. Greater capacity to use research can enhance the former by increasing the effectiveness of public policies and programs, and the latter by providing an independent and scientifically verified rationale for decision-making [42, 45, 46]. In this study, we use the term ‘research’ broadly to include collections or analyses of data, or theory, found in peer-reviewed papers, technical monographs or books, or in grey literature such as internal evaluations and reports on authoritative websites, or presentations or advice from researchers.

Multiple dimensions of capacity affect the use of research in policy-making. At the most concrete level, policy-makers must be able to get hold of useful research. This is research that (1) addresses policy problems, including costs and benefits, and produces findings that can be applied to local decision-making; (2) is accessible, i.e. readable, timely and easy to get hold of (not buried in information tsunamis or located behind firewalls); and (3) which has policy credibility, i.e. is conducted with sufficient scientific rigour to render it reliable, but is also methodologically fit-for-purpose and communicates the findings persuasively [47–49]. Thus, there is a scope to enhance the conduct, presentation and synthesis of research itself, as well as the means by which policy-makers access it [50].

Policy-makers may also need specialist knowledge and skills to access, appraise, generate and apply research in their work. Although many have substantial skills and experience in these areas, others do not [51]; they lack confidence and want training [46, 52]. Individuals’ beliefs about the value of research and requirements of different policy roles are also considered to be important mediators of use [50, 53].

Organisational capacity can constrain or enhance research use, irrespective of individual capabilities [54]. Institutional infrastructure, resourcing and systems, leadership and the underlying workplace culture, have an enormous impact on practice norms and expectations, and opportunities for skills development and application [50, 54–57]. Organisational culture is notoriously hard to access and transform [58], but is considered to be a fundamental indicator, and facilitator, of research-informed practice [42, 59]. This meso level is, in turn, impacted by the wider institutional systems in which policy organisations operate [51]. For instance, dominant views about what forms of evidence are valued and how they are sanctioned or incentivised in policy processes shape what capacities are needed and how they operate, but this sphere remains largely outside the scope of intervention trials [41, 42].

The quality of the relationships between policy-makers and researchers is also (and increasingly) seen as critical for improving the development and uptake of useful research for policy-making [32, 60–62]. Here, capacity-building focuses on forging or enhancing connections across a spectrum of interactivity from information exchange forums to formal partnerships and the co-production of research [63–65]. Individual, organisational and institutional capacity have crucial roles to play in forming and sustaining interpersonal networks [42, 50].

These dimensions of capacity indicate the breadth and complexity of the capabilities that interventions can address, concerned as they are with products, resources, skills, beliefs, values, systems, institutional structures, boundaries and relationships, most of which have interdependencies.

### Aims

This review explores a range of interventions designed to build capacity for research use in policy processes. Outcomes of interest are those related to capacity to use research, including capacity to access and apply research, to work productively with researchers and intermediaries such as knowledge brokers, the establishment of workforce and infrastructure supports, intention to use research and actual use.

Our purpose is two-fold – first, to describe the main characteristics of the interventions, namely the study designs, intervention goals and strategies, implementation settings, participants and outcomes. Second, to consider how process effects and outcomes were generated in those settings (see next section for definitions of these terms) drawing on theory from other studies to develop tentative hypotheses that might be applicable across varied intervention sites. Understanding context and theory is essential for understanding how interventions function in general [66–68], and how research is mobilised [69–71]. Our aim is to provide a first step towards developing practical and theoretically grounded guidance for future interventions of this type. The research question addressed is: What causal mechanisms can best explain the observed outcomes of interventions that aim to increase policy-makers’ capacity to use research in their work? Note, by ‘intervention’ we mean a purposeful attempt to bring about some identified change, this may involve the use of one or more ‘strategies’. We use the term ‘theory’ broadly to encompass a range of formally investigated and informal hypotheses about how intervention strategies bring about change, or why they do not.

### Our approach: a realist scoping review

This a realist scoping review. In general, scoping reviews are “*… a form of knowledge synthesis that addresses an exploratory research question aimed at mapping key concepts, types of evidence, and gaps in research related to a defined area or field by systematically searching, selecting, and synthesizing existing knowledge*” ([72], p. 129–4). In this case, the research question and synthesis were strongly informed by realist philosophy and realist review methods; however, it does not fully adhere to the current criteria for conducting a realist (or rapid realist) review – hence the hybrid term (see Additional file 1 for comparison of scoping reviews, realist reviews and our methodology).

A realist approach is used because we aim to produce findings with potentially transferable implications for the design, implementation and evaluation of other interventions in this field. Realist reviews attempt to identify patterns that are articulated at the level of ‘middle range’ or program theory. This is thought to be most useful for our purposes because it is specific enough to generate propositions that can be tested, but general enough to apply across different interventions and settings [73–75]. Realist approaches are methodologically flexible, enabling reviews of broad scope suited to addressing the complexity of interventions in organisational systems [76], and for addressing questions about how interventions work (or not) for different people in different circumstances [77]. This inclusive approach enabled us to capture studies that used innovative and opportunistic strategies for enhancing research use in policy, and diverse methods for evaluating these strategies.

### Mechanisms, process effects and outcomes

Realist evaluations and syntheses develop and test hypothesised relationships between intervention strategies, implementation contexts, causal mechanisms and observed outcomes. However, contexts, mechanisms and outcomes are not fixed entities in a tidy causal chain, but can shift position depending on the focus of the inquiry, i.e. they function as a context, mechanism or outcome at different levels of analysis and in different parts of a program [31]. For example, if the outcome of interest is research utilisation, then increased capacity is likely to be an important mechanism; however, if the inquiry takes capacity itself as the outcome of interest (as we do in this review), the focus will be on the more granular mechanisms that built this capacity. Therefore, in this review, we are looking for mechanisms that cause, and thus precede, capacity development. Different foci, and the corresponding shift in where we look for elements of causality, have implications for the review and synthesis of intervention studies, as we now explain.

Where interventions are new, or newly adapted, causal relationships are often examined at a relatively detailed level of granularity that emphasises specific points in the causal pathway. Broadly, we see process evaluation, formative evaluation and much of the qualitative research that is conducted in trials as trying to identify and explain how intervention strategies bring about process effects. These are the range of immediate responses (ways of interacting with and ‘translating’ the intervention) that shape distal responses to the intervention (final outcomes).

Intervention designers have hypotheses about what process effects are needed to achieve desired outcomes. Often, these are not articulated, sometimes because they are obvious (e.g. if no one attends a workshop then it clearly cannot be successful), or because these interactions are subsumed in blanket terms like ‘engagement’ and ‘participation’, which mask crucial details about *how* people engaged and participated, or *why* they did not. For example, intervention studies often report on the importance of ‘champions’, i.e. members of an organisation who actively promote an intervention or practice change. When looking at an intervention study as a whole, championing may function as a causal mechanism in that it helps to bring about change, but from a granular perspective, championing can be conceptualised as a process effect because (1) effective championing mediates intervention outcomes (the influence of champions on organisational change initiatives is well documented), and (2) it is generated by interactions between the intervention, participants and context (i.e. it is *caused* – people make conscious judgements about acting as champions). Consequently, in order to inform implementation improvements, and program improvement and adaptation, it may be useful to understand the causes of championing in more detail, for example, by asking ‘In this intervention and context, what perceptions and considerations influenced who became a champion and who did not?’. At this level of analysis, process effects are treated as proximal outcomes. We explore this in more detail elsewhere [78].

**Fig. 1:**
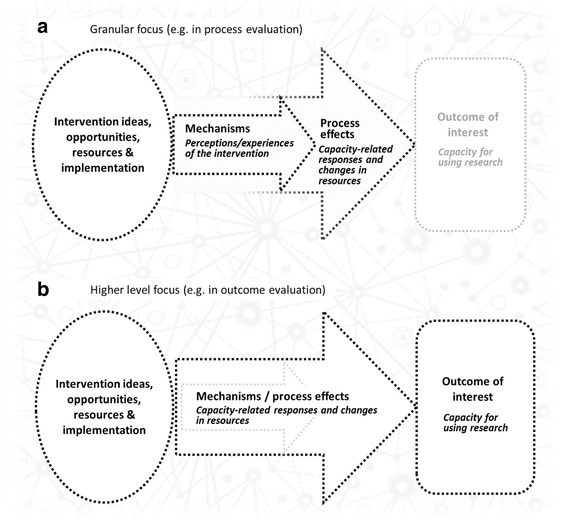
Different levels of focus depending on the evaluation purpose and outcomes in studies of research utilisation capacity-building interventions. The black dotted lines reflect the focus of enquiry. In (**a**), the focus is on immediate responses to the intervention, namely process effects and the mechanisms through which these are brought about. In (**b**), where the focus of the inquiry is on more distal outcomes using a higher level of analysis to investigate causality, mechanisms and process effects are functionally the same thing, i.e. proximal responses to the intervention

Figure 1 depicts how these two levels of focus might be applied to a research utilisation capacity-building intervention. Figure 1a illustrates a granular approach where the evaluation focuses on the relationship between immediate perceptions and experiences of the intervention (which function as mechanisms in this scenario) and how they lead to process effects (capacity-related responses such as participation in skills development programs, relationship development with researchers, or managers funding access to research databases and other workplace supports). This contrasts with Fig. 1b, which depicts an evaluation that is more focused on distal outcomes and thus takes a higher-level perspective, collapsing the causal detail and blurring the distinction between process effects and mechanisms. From this perspective, many process effects are indeed mechanisms.

In practice, this distinction between granular and high-level foci is usually a question of emphasis rather than a demarcation, and it is not always clear where the focus of an evaluation lies. Although realist findings are often tabulated, which can imply that phenomena exist in strict compartments, the propositions that describe causal pathways tend to suggest greater fluidity and often incorporate process effects and the mechanisms that generate them.

We highlight this distinction here because our findings depend on both the models above. This reflects the different evaluative emphases of the reviewed studies, and their diverse outcome measures. Consequently, we present findings that include intervention strategies, contexts, mechanisms, process effects and outcomes.

## Methods

### Focus of the review: what and who?

This review is founded on the realist assumption that the success or failure of an intervention depends on the interactions between the intervention strategies and the implementation context. Identifying patterns in these interactions enables us to develop tentative theories about what may plausibly occur in other interventions [73, 79]. Thus, in reviewing the literature, close attention must be paid to the characteristics of the intervention, the people it targets and the settings in which it takes place. To this end, we differentiate between two types of intervention that are often conflated in the literature. This review focuses on *research utilisation* interventions that aim to increase the effectiveness with which professionals engage with research and use it in their decision-making. We see these interventions as having very different goals to *research translation* interventions that attempt to modify clinical practice in line with research-informed standards or guidelines. The former will generally attempt to build capacity in some form (e.g. by providing resources, training, reflective forums or partnership opportunities designed to enhance professional practice) and may increase individuals’ agency and critical engagement with research, whereas the latter more often seek to institutionalise adherence to a specific practice protocol that may constrain autonomy and critical reflection. Harrison and McDonald [80] make a similar distinction between the “*critical appraisal model*” of research-informed practice, where participants are encouraged to critique and incorporate concepts in their practice, and the “*scientific-bureaucratic model*” that attempts to regulate practice. The contrasting ‘politics of knowledge’ inherent in these two models are likely to trigger quite different responses in relation to professional identity, self-determination and organisational accountability [38, 80].

Second, we differentiate between research utilisation capacity-building interventions targeting policy-makers in government agencies and those targeting health practitioners based in service organisations. While both tackle complex systems with distributed decision-making, we believe that the contextual characteristics of bureaucracies differ from those of clinical practice in ways that may affect the functioning of interventions. This is especially pertinent for interventions that attempt to build research utilisation capacity because the forms of research that are most useful in these contexts are likely to differ. For example, biomedical and clinical research (including randomised controlled trials) may be more useful in healthcare settings, whereas health policy-makers might be better informed by research that incorporates scaled analyses of effectiveness, reach and costs [47]. Thus, studies included in this review are limited to research utilisation capacity-building interventions that target policy-makers.

### Search strategy

**Fig. 2:**
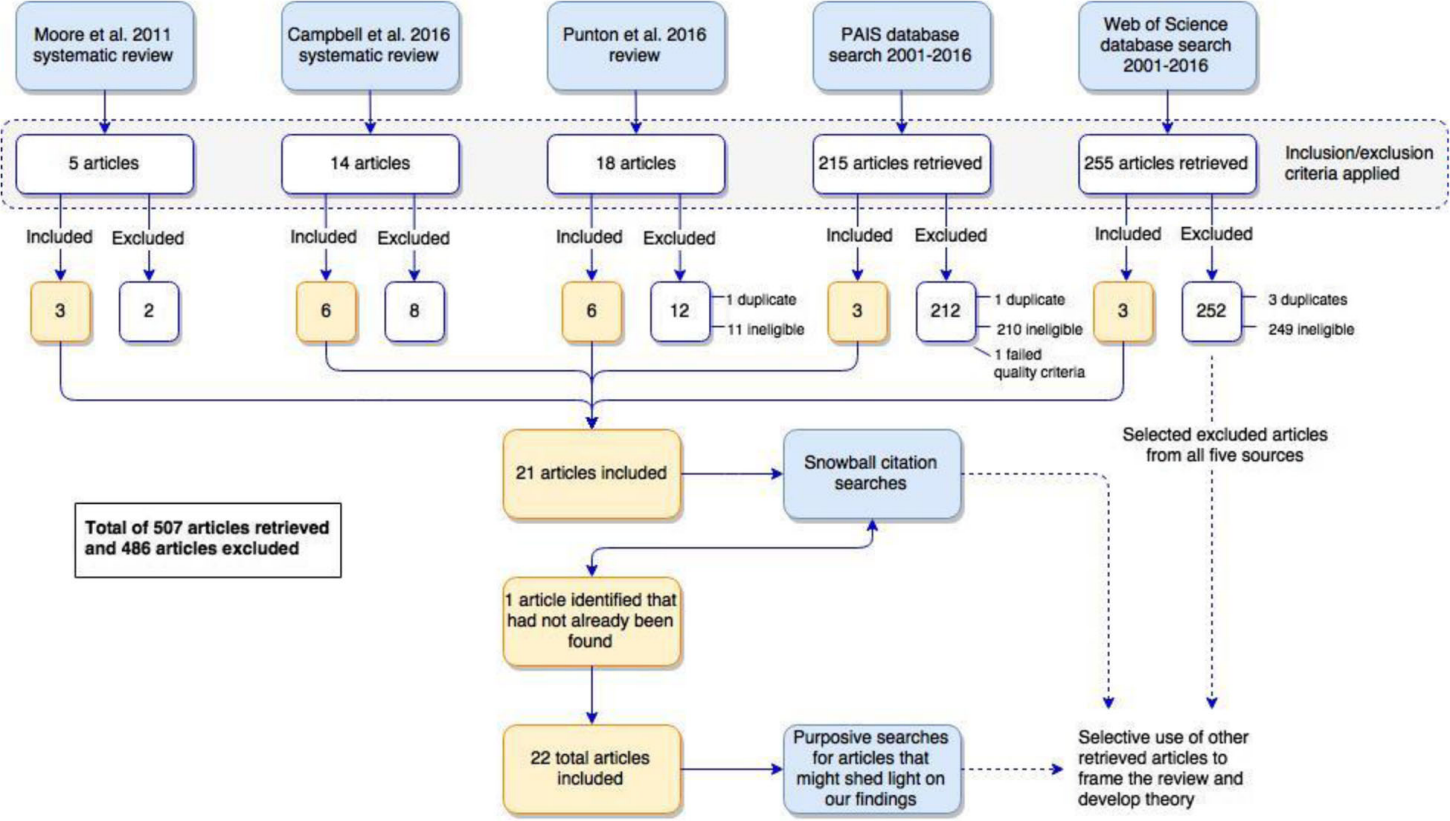
Review search strategy

The literature encompassed in this review was identified in three ways:

1. From three existing reviews of published peer-reviewed papers that reported on the evaluation of strategies aimed at increasing the use of research by decision-makers in public policy and program development. The first two reviews are complementary; one [81] captured papers published between 1999 and 2009, while the other [82] captured papers published between 2009 and 2015. These reviews focus on identifying the strategies employed to increase research use and the “*factors associated with these strategies that are likely to influence the use of research*” [82]. The third review [42], conducted for the United Kingdom’s Building Capacity to Use Research Evidence program, focused on primary intervention studies aimed at developing capacity for research use in public sector decision-making. It included studies published between 2003 and 2014. These sources were deemed to have identified most relevant peer-reviewed papers pertaining to the testing of research utilisation interventions in policy agencies between 2001 and 2015.
2. From two searches on academic databases: one on PAIS and the other on Web of Science (WoS), searching articles published between 2001 and 2016. See Additional file 1 for the search syntax and filters used, and the rationale for selecting these databases.
3. Iterative searches on Google and Google Scholar using keywords and snowballing from citations in previously found papers, reports and journal articles. These were exploratory rather than exhaustive searches, intended to identify papers, reports and commentary that would increase our awareness of diverse perspectives that, in turn, could help us draw theoretically grounded lessons from the intervention studies identified in steps 1 and 2 above. Figure 2 depicts the search and exclusion strategy used.

### Inclusion criteria

Studies were included provided they met the following criteria:

- **Interventions.** The study reported on an intervention designed to improve or increase policy-makers’ capacity to use research in their work. We took a broad view of capacity-building that included strategies for supporting research use as well as strategies for advancing it, so studies were included if they were designed to enhance access to research; skills in appraising research, generating research (commissioning or conducting it) or applying research; organisational systems for developing and supporting research use; and/or connections with researchers (including partnership work and co-production). Any strategy that employed one or more capacity-building activities aimed at individual-level or organisational structures and systems was eligible, irrespective of whether the strategy was part of a research study or initiated/implemented by policy agencies, or a combination. Studies were excluded if they focused on policy development capabilities in which the use of research was a minor component (e.g. [83]); evaluated one-off attempts to get policy-makers to use research for a specific policy initiative; or focused on specific fields other than health such as education or climate change (e.g. [84]). Studies that addressed the use of research by policy-makers in general were included (e.g. [41]).
- **Populations.** The intervention targeted policy-makers, by which we mean either (1) non-elected civil servants working in some aspect of policy or program development, funding, implementation or evaluation within government agencies such as Departments of Health, and/or (2) senior health services decision-makers, e.g. executives in regional or federal health authorities who have responsibility for large scale service planning and delivery, and/or (3) elected government ministers. We included studies where participants included policy-makers and other groups (e.g. frontline clinicians, NGO officers or researchers) and where some disaggregated data for the different groups was reported, but not those in which intervention effects/outcomes were reported in aggregated form, for instance, health staff at all levels took part in the intervention but the data for senior health services decision-makers was not reported separately from that of frontline staff (e.g. [25, 85–91]).
- **Study design.** The intervention was evaluated. This includes process evaluations and reports of ‘soft’ proximal outcomes such as satisfaction and awareness, which we conceptualise in our analysis as mechanisms. As mentioned, opportunistic evaluations of initiatives that were planned outside the auspices of a research study were included, but studies were excluded if they described achievements or ‘lessons learnt’ but did not explain how such information was captured as part of an evaluation strategy (e.g. [32, 84, 92–94]).
- **Publication.** The evaluation results were published in English between 1999 and 2016. This date range was judged by the authors as likely to encompass the vast majority of relevant publications in this field, and included the earliest relevant study of which we were aware [95].
- **Settings.** We included studies from all countries, including low- and middle-income countries. These settings are likely to differ considerably from those of high-income countries (e.g. less developed infrastructure, fewer resources, different health priorities and a poorer local evidence-base [96]) but, together, the studies provide insights into creative interventions and produce findings that may have implications across the country income divide in both directions.
- **Quality.** Studies were excluded if they were “*fatally flawed*” according to the appraisal criteria used in critical interpretive synthesis [97]. Following Dixon-Woods et al. [97], we used a low threshold for quality pre-analysis due to the diversity of methodologies in our final sample, and because our method of synthesising data would include judgements about the credibility and contribution of studies.

### Analysis

We took an inductive approach to analysis (e.g. [45]) guided by realist thinking rather than starting with an a priori framework or conceptual categories. This was due to the breadth of strategies we were investigating and their diverse theoretical implications. As befits an exploratory realist review, our aim was to identify “*initial rough theories*” that might explain how and why different strategies worked (or did not) within those intervention settings [75]. While none of the studies are realist themselves, many pay close attention to context, process and interaction, so there is some rich information with which to start developing tentative hypotheses about how and why the interventions had the effects they did [75, 98].

These terms are used in accordance with the realist movement associated with the United Kingdom’s RAMESES projects, which aim to produce quality and publication standards for realist research [99].

Following Best et al. [100] a six-step process was used in which we (1) read and reread the 22 articles to gain familiarity with the data; (2) extracted details about what the intervention comprised, contextual features and the studies’ empirical findings, including any clues about interactions and causality; (3) extracted program theory made by authors of these studies, i.e. explicit or inferred hypotheses, concepts or principles about what the intervention was expected to do or how it was expected to work [75]; (4) reviewed the intervention strategies, findings and theories of related intervention studies that had similar aims but did not meet our inclusion criteria; (5) identified further relevant literature that might help develop new theories and/or additional detail, including papers cited by authors of the primary studies and literature from fields that were likely to shed light on or challenge our findings (time and resource constraints limited this step, so it was pragmatic rather comprehensive, drawing on materials known to the authors as well as found articles); and (6) summarised the connections we inferred between intervention strategies, implementation contexts, underlying casual mechanisms and observed outcomes in strategy + context + mechanism = outcome (SCMO) configurations (this was an iterative process that repeatedly cycled back through the activities described above). Table 1 describes how the main concepts were defined and identified in this process.

**Table 1:** Definition and identification of concepts in SCMO configurations

Concept	Definition of concept in this review	How this was identified in the analysis
Intervention strategy	Intervention strategies work by changing participants’ reasoning and resources; importantly, they do not work in a void, but interact with contextual features to generate change	The intervention strategies listed in the results were identified from authors’ descriptions of attempts to support or advance policy-makers’ capacity to use research in their work (listed by study in Additional file 2)
Context	Context is any condition that affects how people respond to intervention strategies (i.e. if and how mechanisms are activated); they include settings, structures, circumstances, and the attributes and attitudes of those delivering and receiving the intervention	Contextual features were identified primarily from authors’ accounts of intervention settings and circumstances before and during implementation. On occasion, they were inferred from information about responses to the intervention. In the SCMO tables that follow, we focus on aspects of context that relate specifically to each mechanism; a more general overview of context is provided first
Mechanism	Mechanisms are how an intervention works; they are responses to the resources, opportunities or challenges offered by the intervention. Mechanisms are activated to varying extents (or not at all) depending on interactions between intervention strategies and contexts. Although mechanisms are not observable, their impacts are, so they can be inferred, tested and refined	With one arguable exception [101], none of the studies explicitly identified or tested causal mechanisms so we inferred mechanisms from authors’ accounts of how the intervention was conceived, designed, delivered and received (i.e. how it was meant to function and how it actually functioned); this was supplemented with similar information from some of the non-eligible studies that were identified in this search, and from the wider theoretical and implementation literature. Mechanisms posited in the results tables that follow include hypotheses about (1) how each intervention strategy worked (where mechanisms were activated successfully) and (2) how strategies would have worked if they had been appropriate in that context (where mechanisms were not activated and their absence may account for poor outcomes)
Outcome	These are intended or unintended impacts generated by mechanisms; as described in the previous section, in this review outcomes may be proximal (process effects) or more distal study outcomes	Outcomes of interest were explicit in most of the reviewed studies; where they were vague, we inferred them from the studies’ research questions, interview foci and reported results ( described in Additional file 2)

AH led this process. SB read the synthesis of studies and critiqued the draft SCMO configurations. SJR independently read the included studies, made notes in relation to steps 2 and 6, and critiqued the revised configurations. AH and SJR iteratively workshopped draft findings to reach agreement, drawing on feedback from our co-authors to resolve remaining areas of debate and to refine the final SCMO configurations.

## Results

### Search results

As Fig. 2 shows, 5, 14 and 18 articles, respectively, were identified from the three reviews. Of these 37 articles, 15 met our eligibility criteria. Another 3 articles were identified from the PAIS search, and 3 from the WoS search. A further article was identified from citation snowballing, resulting in 22 included studies. One article from the PAIS search was excluded on quality criteria as it provided so little detail of the intervention strategies and evaluation methods that we could not see how the conclusions were reached. Additional file 2 presents a tabular overview of the included studies’ aims, design, intervention strategies, participant numbers and characteristics, context, evaluation methods, outcome measures, and key findings. This table includes theories, models or frameworks mentioned in the article as informing the design of the intervention or evaluation.

### Study design

Study design terminology is often inconsistent. Here, we use three terms to describe the overarching study design – ‘experimental’ indicates that the research team provided the intervention and evaluated it using some form of randomisation and control groups; ‘interventional’ that the research team provided the intervention and evaluated it, but not using experimental methods; and ‘observational’ that the intervention or initiative being evaluated was not designed as part of a research study. Based on these definitions, 12 of the studies appeared to be observational, seven interventional, and three experimental. These included process evaluations and opportunistic evaluations of projects, services or strategies that had been initiated by others.

Outcomes of interest were diverse, ranging from activities that can be measured objectively, such as increased use of systematic reviews [95], to more conceptual outcomes such as greater strategic use of data [101, 102], ‘catalysing’ research use [103] and fostering a culture of critical thinking [104].

#### Domains of capacity-building and support

Eighteen strategies for supporting or increasing capacity were identified within four non-exclusive domains of research use capacity. These were ‘Access to research’ (16 studies); ‘Skills improvement’ in accessing, appraising and/or applying research in policy work (13 studies); ‘Systems improvement’ tackling organisational or cross-organisational infrastructure, processes and/or resources (8 studies); and ‘Interaction’ with researchers (11 studies) (Table 2).

**Table 2:** Research utilisation domains and strategies used within the reviewed studies

Research utilisation domain	Intervention strategies (and number of studies that used it)
Access to research	1. Providing access to research articles or syntheses via an online database (5) 2. Disseminating tailored syntheses summaries or reports, including policy briefs (7) 3. Commissioning research and reviews (2) 4. Seminars or other forums in which research findings are presented (4) 5. Facilitated access using a knowledge broker or other intermediary (3)
Skills improvement	6. Skills development workshops (10) 7. Intensive skills training programs (4) 8. Training or support for managers in championing and modelling research use (4) 9. Mentoring (includes using knowledge brokers to build skills) (7) 10. Goal-orientated mentoring (with presentations or assessment) (4)
Systems improvement	11. Improving infrastructure, e.g. library, new research portals, data sharing software (5) 12. Improving organisational tools, resources and processes, e.g. procedures, toolkits, knowledge management protocols, funds for commissioning research (2) 13. Workforce development, e.g. research-related positions and incentives (1) 14. Establishing internal research support bodies, e.g. research units and committees (3)
Interaction	15. One-off or periodic interactive forums, e.g. roundtables, cross-sector retreats, policy dialogues (4) 16. Platforms for ongoing interactivity, e.g. community of practice, cross-sector committees (4) 17. Collaboration in the development of a research report or policy brief/dialogue (2) 18. Partnership projects: research co-production (3)

Most evaluations used case study methodologies. The main data collection methods were interviews (14 studies), focus groups (6 studies) and questionnaires (11 studies: 6 cross-sectional post-intervention and 5 pre/post); therefore, outcomes were largely self-reported. Two studies reported on the validity of their survey instruments. Eight studies also reviewed relevant documents, two conducted a network analysis, and one used observations of the intervention activities. Three studies used independent experts to assess intervention outputs. Participation data such as attendance rates at workshops and numbers of participants in the intervention and evaluation were reported to varying extents. The nine studies that included statistical analyses had diverse outcome measures, and used different sampling, data collection and modelling methods. See Additional file 2 for further details on aspects of study design.

Two whole-of-intervention design strategies were also widely used, namely needs-based tailoring (10 studies) and multi-component programs (17 studies) (Table 3).

**Table 3:** Focus of intervention studies targeting research utilisation in policy-making 2001–2016

Study reference (chronological order)		Intervention strategies as listed in Table 2	Intervention domains				Intervention design	
			*Access*	*Skills*	*Systems*	*Interaction*	*Needs-based tailoring*	*Multi-component*
1.	Dobbins et al., 2001 [95]	2	✓					
2.	Pappaioanou et al., 2003 [102]	1, 7, 8, 9, 11, 12	✓	✓	✓		✓	✓
3.	Kothari et al., 2005 [91]	2, 17	✓			✓		
4.	Dobbins et al., 2009 [106]	1, 2, 5	✓		✓			✓
5.	Wehrens et al., 2010 [138]	4, 18	✓			✓	✓	✓
6.	Brownson et al., 2011 [202]	2	✓				✓	
7.	Campbell et al., 2011 [29]	3, 5	✓				✓	
8.	Rolle et al., 2011 [101]	7, 8, 9, 10		✓				✓
9.	Peirson et al., 2012 [104]	3, 6, 11, 12, 13, 14	✓	✓	✓		✓	✓
10.	Uneke et al., 2012 [132]	6		✓				
11.	Dagenais et al., 2013 [129]	2, 4, 15	✓			✓		✓
12.	Hoeijmakers et al., 2013 [127]	6, 11, 18	✓	✓	✓	✓	✓	✓
13.	Waqa et al., 2013 [134]	1, 6, 9, 10, 11	✓	✓	✓		✓	✓
14.	Kothari et al., 2014 [62]	16, 18				✓		✓
15.	Traynor et al., 2014 [133]	1, 2, 5, 6, 8, 9, 12	✓	✓	✓			✓
16.	Brennan et al., 2015 [110]	1, 6, 16	✓	✓		✓		✓
17.	Dwan et al., 2015 [128]	4, 15	✓			✓		✓
18.	Shroff et al., 2015 [103]	2, 6, 14, 15	✓	✓	✓	✓	✓	✓
19.	Uneke et al., 2015 [130]	6, 9, 10		✓				✓
20.	Uneke et al., 2015 [137]	6, 7, 8, 9, 10, 16, 17		✓		✓		✓
21.	Hawkes et al., 2016 [41]	4, 6, 11, 14, 15	✓	✓	✓	✓	✓	✓
22.	Langlois et al., 2016 [131]	7, 9, 16		✓		✓	✓	✓

### Intervention participants and settings

All studies targeted capacity in policy-makers and/or policy agencies in that they were attempting to support, increase or otherwise improve policy-makers’ access to research; policy-makers’ skills in accessing and/or using research; the capacity of systems in policy organisations to support research use; and/or interactions between researchers and policy-makers that were intended to facilitate knowledge exchange or partnership work. Many studies had more than one category of participant, e.g. a mix of policy-makers, practitioners and researchers. The majority included bureaucrats in government departments of health or equivalent at the regional level (11 studies) or national/international level (9 studies). Eleven studies targeted government employees running regional health services, and two included elected (ministerial) policy-makers.

The intervention settings spanned 18 countries. There were 17 single-country studies conducted in Canada (*n* = 5), Australia (*n* = 3), Nigeria (*n* = 3), The Netherlands (*n* = 2), Burkina Faso (*n* = 1), Ethiopia (*n* = 1), Fiji (*n* = 1) and the United States of America (*n* = 1). Four were multi-country studies were conducted, respectively, in Bangladesh, Gambia, India and Nigeria; Cameroon and South Africa; Bolivia, Cameroon, Mexico and the Philippines; and Argentina, Bangladesh, Cameroon, Nigeria and Zambia. Finally, one was an international collaboration run from Canada. Thus, 10 studies took place within one or more low- and middle-income country [105].

### Program theories

The 22 studies draw on a diverse suite of theories and concepts. None present a formal program theory, but many use frameworks to guide intervention development. The RCT conducted by Dobbins et al. [106] is based on diffusion of innovations [107–109], Brennan et al. [110] use the theoretical domains framework [111], and Shroff et al. [103] adapt a knowledge translation framework [45]. Others draw eclectically on concepts from a variety of sources, including (1) studies of how research is used in policy-making – both systematic reviews [49, 112] and individual studies such as Weiss’s seminal typology of research utilisation [113]; (2) models for mobilising research such as knowledge transfer frameworks [114–116] and partnership approaches [61, 64, 117–119]; (3) analyses of barriers to researcher–policymaker relationships [53, 120] and ‘gap-bridging’ solutions such as the linkage and exchange model [121], and the use of knowledge brokers [122]; (4) studies of organisational support for research use [59, 123]; (5) guidance for facilitating the use of research in policy-making, including in low- and middle-income countries, e.g. the SUPPORT tools developed by Oxman et al. [124, 125] and Lavis et al. [126]; and (6) WHO commissioned reports on building capacity for health policy and systems research [50, 54].

A minority of studies developed their own program theory or conceptual framework that guided the intervention design and evaluation (e.g. [91, 102, 127]), and some report using frameworks primarily for the evaluation (e.g. [41, 101, 104]).

### Intervention strategies, contexts, causal mechanisms and outcomes

The findings derived from our realist analysis of the 22 studies are now presented. See Additional file 2 for a summary of each study’s design, outcomes and informing theory.

#### Overarching contextual considerations and their implications

Nearly all the studies in this review conceptualised research use as contextually contingent. They assumed that some degree of responsivity to local needs was required by research providers, and that policy-makers’ judgements about the usefulness of research were flexible, according to shifting circumstances, and based on far broader criteria than academic hierarchies of evidence, e.g. “*Research is only as useful as potential users perceive it to be, irrespective of its methodological rigour or its findings’ power*” ([128], p. 241). Some pointed to the limitations of technical-rational models of research use in political decision-making [95, 102, 128], and over half emphasised the complexity of policy-making [29, 41, 62, 102–104, 106, 110, 127, 129–131]. Terminology reflected the acceptance that policy will never be based entirely on research [5]. Indeed, few of the studies used the term ‘evidence based policy’ unquestioningly, preferring more nuanced post-evidence based terms such as ‘evidence-informed policy’ [103, 132], ‘evidence-informed decision making’ [104, 106, 133], and ‘research-informed policy’ [29, 110, 134].

From our analysis, it appeared that there were some similar contextual factors in all the studies reviewed, despite their very different settings (e.g. Burkina Faso and Canada). This suggests there may be universal influences on the use of research in policy-making of which virtually every capacity-building intervention should take account. The main contextual factors identified were:

- **Research characteristics:** Policy-makers’ use of research was influenced by the degree to which they were able to obtain research that was relevant, applicable and easy to read. This suggests the need for strategies that increase the availability and accessibility of fit-for-purpose research findings. Credibility of research and researchers is also a consideration.
- **Individual characteristics:** Policy-makers’ use of research was affected by existing research-related knowledge, skills and self-confidence, and views about the value of research. The former clearly indicates task-appropriate skills development and support, but the latter suggests that, in some cases, it will be necessary to influence beliefs.
- **Interpersonal characteristics:** Although neither community is heterogeneous, there were common differences between policy-makers and researchers in terms of language, values, expectations and incentives. This suggests the need for strategies that either bridge these communities, or form more cohesive connections between them, potentially blurring their boundaries.
- **Organisational characteristics:** Research use in policy agencies was shaped by organisational culture. Agency remits, resources and constraints further influenced how research was prioritised and used. This suggests the need to activate structural mechanisms that increase expectations of, and facilitate, research use in day-to-day practice. Underlying values and assumptions may need to be influenced. Leadership by managers and opinion leaders is likely to be key.
- **Environmental characteristics:** Policy-making environments were complex, political and responsive, affecting what research could be used for what purposes, and the time available for this. The way that research is (or can be) interpreted in this argumentative arena is likely to determine its role in policy processes, thus relevance, applicability and credibility are not fixed research characteristics but determined in relation to circumstances. This suggests that tailored research (both in terms of methodology and presentation), rapid production of findings and responsive dialogue with researchers may be valuable. Methods for supporting this are likely to include commissioning and/or research-policy partnerships and/or internal generation of research by policy agencies.

These overarching contextual factors align with other reviews, which conclude that policy-makers’ capacity and motivation to use research is shaped by forces such as these at micro, meso and macro levels [43, 49, 112].

The next four sections present our findings in relation to the four domains of capacity previously identified. As per the focus of this review, the emphasis in these results is not on the extent to which interventions were successful in effecting change, but on how change was effected or why it was not; consequently, the narrative overview in each of the results sections is on mechanisms. The tables that follow place these mechanisms in context by showing what intervention strategy was used; key contextual factors identified in the studies; possible causal mechanisms including those that appear to have been activated in the studies and those that were apparently required but were not activated; and any reported results relating to that strategy (including process effects and study outcomes) [73, 135]. Our hypotheses describe mechanisms that were inferred from multiple studies and/or powerfully evident in one study, and are supported by theoretical or empirical implementation literature. Where low effects are observed, we hypothesise that one or more key mechanisms were not activated, or were activated too weakly to bring about the level of desired change. Note that the contextual factors described above are considered to be ‘givens’ in these tables and so are only reiterated where they seem to be most crucial.

### Access to research (Table 4)

Mechanisms that appeared to underpin access to online research included awareness of resources and the relative merits of different kinds of research within them; valuing what was on offer; the efficiency with which research could be obtained; and confidence in using resources and their contents. When research was synthesised, tailored for specific users, and sent to them, ease of access and ease of use aided uptake, probably aided by increased policy-relevance and applicability. As with all domains, perceived fit between what was offered and policy needs/priorities was key. The tailored, contextualised and inclusive evidence synthesis provided by evidence briefs tick many of these boxes. Commissioning rapid reviews maximised policy-makers’ engagement with and control over the research focus, methods and timeliness. The costs and process of commissioning are likely to increase investment in using the end-product.

The value of seminars seemed to be enhanced by tailoring and interactivity, and by the credibility and communicative skills of the presenters who engage policy-makers despite the often dry content. Meeting researchers at these seminars can break the ice and lead to further interaction.

Intermediaries such as knowledge brokers can facilitate access to research by providing navigational support in unfamiliar terrain. They provide a communicative bridge by helping policy-makers articulate needs and expectations and, in some cases, translate these for researchers. The intermediaries’ interpersonal skills, credibility, availability, ability to provide individualised support and perceived neutrality enabled the relationship to work, but this also requires time in less research-orientated settings.

**Table 4:**
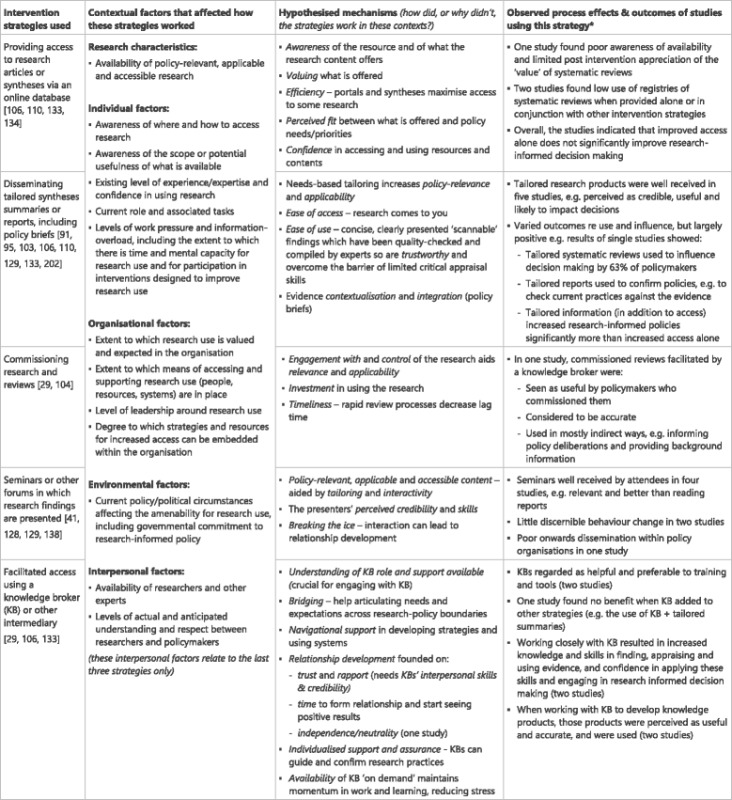
Access to research intervention strategies, context, mechanisms and impacts [29, 41, 91, 95, 128, 129, 103, 104, 106, 110, 133, 134, 138, 202]

*In this and subsequent tables, not all the studies that target each domain will necessarily be included, for example, where a study’s strategies for increasing access was based on skills or systems improvement, or interaction, it is not cited in the table above. Calculations of the number of studies where process effects/outcomes were observed (in the last column) are based only on studies cited in the intervention strategies column

### Skills improvement (Table 5)

Mechanisms for skills improvement in using research appear to include policy-makers believing in the relative advantage of participation, which is affected by the perceived appropriateness/desirability of intervention goals and the relevance, applicability, accessibility and credibility of intervention content. Andragogical principles that emphasise participant-driven learning (manifested in a partnership approach that may include needs consultation, tailored content, informal information exchange and practice opportunities) engages policy-makers. Participants’ active input appears to maintain interest and investment. Strengths-based learning that develops self-efficacy increases motivation and can empower policy-makers to become research champions and train or mentor others. Strong leadership support for the intervention and its goals, including modelling research use practices, is emblematic of wider organisation commitment to using research. Targeted policy-makers will have to find training manageable if they are to attend; this may require pragmatic timing and workarounds.

Mentoring works by providing individualised guidance and support about the real-world application of new knowledge and skills, which in turn increases self-efficacy as abstract learning is turned into concrete practice. Mentors’ credibility and experience, and relationship skills, are crucial. Participants’ accountability, triggered by the need to present their work and/or have it assessed, increases motivation to develop competence in using new knowledge and skills.

**Table 5:**
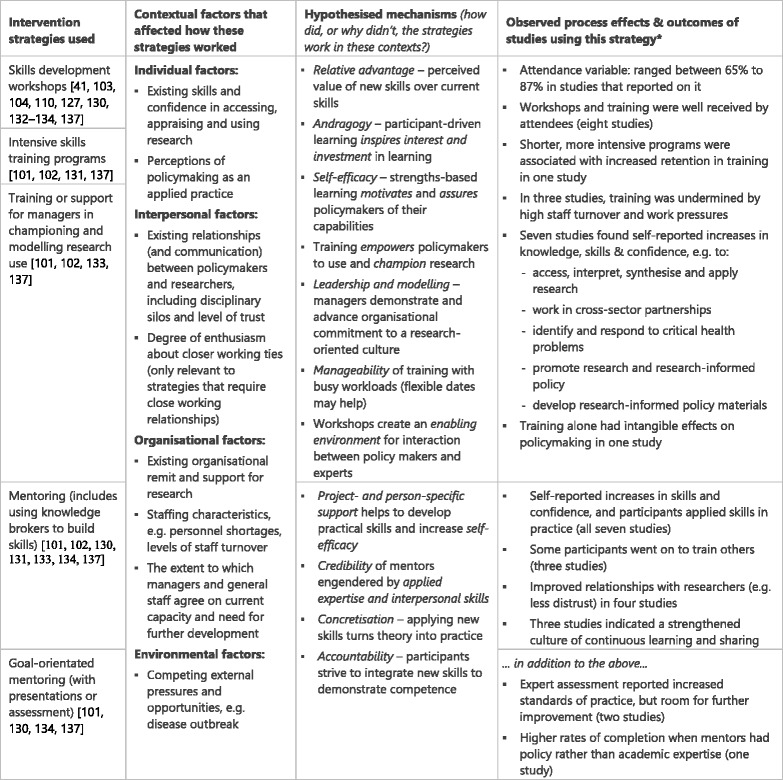
Skills improvement intervention strategies, context, mechanisms and impacts [41, 101–104, 110, 127, 130–134, 137]

### Systems improvement (Table 6)

The mechanisms underpinning systems improvement appear to be diverse, reflecting the breadth of strategies. Diffusion of innovations theory [108, 109, 136] helps makes sense of findings across the studies in relation to interactions between new infrastructure, tools and processes. It posits that new systems must be compatible with key professional and organisational culture and values, flexible enough to accommodate aspects of existing practice that participants will not relinquish, sufficiently easy to use so that policy-makers are not deterred, and have relative advantage, i.e. seem better than existing systems for the individual and for the organisation, so it feels worth the effort of adaptation. Participatory planning and implementation of systems improvement with potential participants may most effectively engage and enthuse them, increasing their readiness for change as well as making the intervention more fit-for-purpose.

Improved systems widen opportunities for using research by increasing ease of access. Where research skills are brought into and developed within the organisation, there is strengthened belief in managers’ commitment to research use. Co-location and control of expertise is likely to increase the policy-relevance, applicability, accessibility and, probably, timeliness of research outputs and advice. In-house research expertise provides opportunities and incentives that policy-makers may find motivating. In general, systems improvements help to embed research use in day-to-day practice and demonstrate managerial commitment, both of which contribute to a research-oriented culture.

**Table 6:**
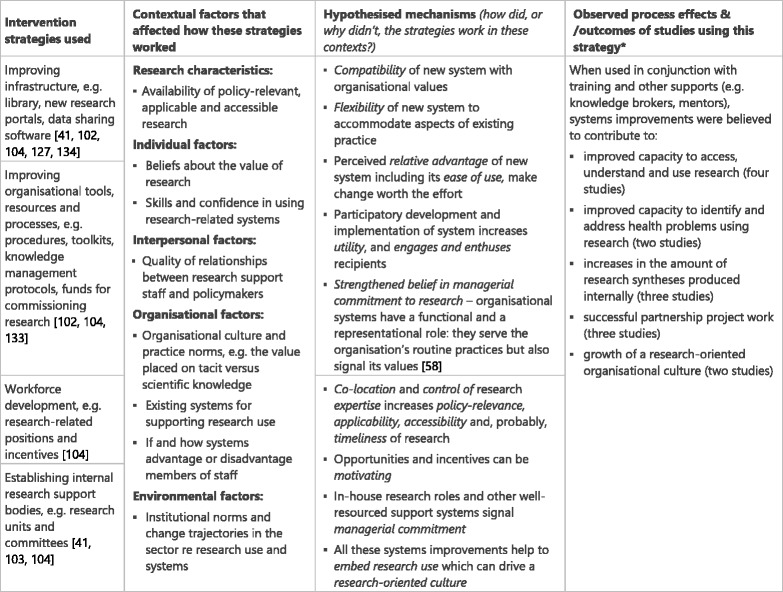
Systems improvement intervention strategies, context, mechanisms and impacts [41, 127, 102–104, 133, 134]

### Interaction with researchers (Table 7)

Mechanisms for productive interactions between policy-makers and researchers appear to include mutual commitment to investing time and effort in interaction, and mutual interest (including the identification of benefits to both parties) in the endeavour. Trust, respect and communicative ease underpin relationship formation, but this takes time to develop and may require repeated interactions. Further, researchers are perceived as neutral, dispassionate contributors.

Where positive interaction is underway it can sensitise and upskill both parties through learning from each other about their values, work contexts and practices. Interactions are more sustainable when there is strong organisational support, and where formal arrangements are put in place rather than relying on individuals (who may move on). Leadership and championing from respected ‘insiders’ may motivate staff to engage with the intervention and put it into practice. Known contacts can act as linkage agents, introducing people to networks and keeping them connected. Collaboration increases ownership of and investment in the research process and outputs, but only when it is genuine, i.e. when both parties have the power and ability to shape critical decisions and have input into processes. However, genuine collaboration is often hard to facilitate. Good governance arrangements can help by ensuring that costs and rewards are agreed and shared, roles are clear, and expectations are articulated and met. Reflexivity, namely paying attention to partnership processes, critiquing and seeking to learn from them, perhaps through developmental evaluation approaches, may combat the lure of traditional silos and disciplinary norms that have been found to undermine collaborations.

**Table 7:**
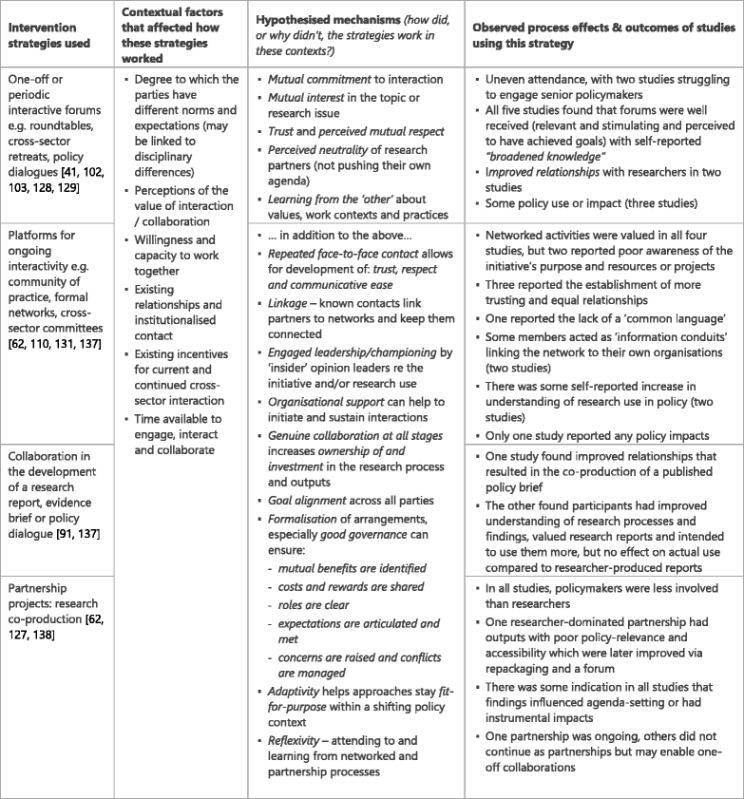
Interaction intervention strategies, context, mechanisms and impacts [41, 62, 91, 102, 103, 110, 127–129, 131, 137, 138]

One of the challenges in evaluating interactive initiatives is the increased entanglement of strategies, mechanisms, process effects and outcomes. For instance, existing positive cross-sector relationships may function as both a context and a mechanism; trust may function as both a mechanism and process effect; and improved relationships may be an outcome while also providing context for further dialogue and partnership work. Thus, there are dual functions and feedback loops implied in much of this theorising.

### Whole-of-intervention design strategies (Table 8)

Although many interventions were described as ‘tailored’, only 10 of the reviewed studies both reported using formal needs analyses or consultative/collaborative strategies for determining needs and preferences and gave some indication of how this shaped the intervention. There was very little information about how this might have impacted responses to the intervention. Nevertheless, it seems likely that tailoring based on accurate needs assessment will maximise the interventions’ compatibility with local needs and practices, and its ability to build on local strengths. Where participants collaborate in tailoring they are more likely to feel like respected partners in the intervention and thus to have ownership of and investment in its outcomes.

As shown in Table 3, most studies that employed multiple intervention strategies did so to improve capacity across two or more domains (e.g. access, skills and interaction) in order to address different levels of support and constraint in research use. Only three studies used multiple intervention strategies to improve capacity in a single domain (e.g. a combination of training workshops, mentoring and practice assessment were used in conjunction to build individual skills). Possible mechanisms are triggered by the interaction and complementarity of multiple strategies, which may be increased when multiple domains are targeted because strengthening capacity in one area (e.g. organisational systems) is likely to support capacity growth in other areas (e.g. individual skills), and strategies may function synergistically to shape a conducive environment for research use. As such, they may both represent and facilitate a culture of research use.

**Table 8:** Key whole-of-intervention strategies, context, mechanisms and impacts

Intervention strategies	Contextual factors	Hypothesised mechanisms	Potential process effects/outcomes^a^
Local tailoring based on needs/situational analysis [41, 102, 131, 133, 134]	Each intervention site has unique features that interact with the implemented strategies [79] Policy-makers have existing strengths and skills; their needs will vary	• Tailoring based on high quality needs/situational analysis maximises intervention compatibility, including its ability to build on local strengths and tackle areas of real need • Where participants collaborate in tailoring they feel respected/heard and have increased ownership of and investment in the intervention outcomes	• Greater acceptance of the intervention’s local fit and utility • Active support of the intervention and its goals
Using multiple strategies to target different forms and levels of capacity [41, 101–104, 106, 110, 127–131, 133, 134, 137, 138]	Research use is multi-factorial Capacity exists in different forms and at different levels (individual, interpersonal, organisational and wider environmental); supports and constraints in one area or at one level affect responses in others	• Strengthened capacity in one area supports capacity growth in other areas • Strategies interact synergistically to shape a conducive environment for research use	• Greater, and more sustainable, change in using research

^a^These outcomes are speculative: there was no clear evidence of outcomes relating to these strategies in the studies

## Discussion

The 22 studies in this review display a diverse suite of theories, concepts and frameworks from different disciplines and fields. We identified 18 intervention strategies and two key design strategies targeting four domains of research use capacity, namely Access, Skills improvement, Systems improvement and Interaction. These studies reflect dominant concerns in the literature about the paucity of policy-usable research and difficulties locating it within information-saturated environments; the need for policy-makers to have adequate skills and confidence in using research, and for work processes and infrastructure to support this use; and the benefits of researchers and policy-makers developing relationships that facilitate mutual understanding, information exchange and collaboration. Underpinning much of the above, are concerns about how the value of research in policy processes is perceived by policy-makers and how these beliefs are affected by organisational cultures.

Despite drawing on ideas from different traditions, most of these studies rejected linear pipeline models in which universally applicable research findings can be ‘transferred’, and favoured more nuanced notions of both the research product and the policy process. Prominent ideas included policy-making as information bricolage in which research findings are only one component (e.g. [41, 102, 103, 106, 137]); the rhetorical and political dimensions of research use (e.g. [91, 128, 129, 138]); and research use as situated – dependent on myriad fluctuating contextual factors (e.g. [103, 110, 128]). Correspondingly, the findings indicated scant instrumental use of research. Indeed, they showed that even where specific research was valued and understood it was seldom translated directly into policy action [29, 91], and that some forms of use did not correspond with established typologies [91].

Like others, we cannot identify one strategy as superior to others in building the capacity of policy-makers to use research [41, 81]. Policy-making is a complex and contingent process, and the various capabilities that facilitate it operate at multiple levels, including the meso and macro levels, where local infrastructures, politics and issue polarisation is likely to impact what is viable [41, 43]. A combination of strategies that are responsive to changing conditions is likely to be most appropriate. Further, regardless of the design features of the intervention, it will be interpreted and enacted differently in different settings [139]. Nevertheless, there are lessons from the 22 studies in this review that have transferable implications for the design and implementation of research utilisation capacity-building interventions in policy agencies, some of which we now discuss.

### Access

It is axiomatic that policy-makers cannot use research if they do not know about it. To this end, efficient routes to relevant, clearly presented research findings are a boon. Tailored and contextualised syntheses – either in document form or via presentations, seminars and advice from knowledge brokers or researchers – seem to offer the most helpful means of providing this access. The benefits of tailoring information for specific audiences and using active dissemination are supported by other reviews [12].

While these studies clearly demonstrated the importance of research being policy relevant, applicable and credible, these concepts raise problems of their own. Regarding credibility, participants did not always judge the merits of research using academic hierarchies of evidence. Weiss suggests this is because policy-makers assess research credibility using ‘truth tests’ (are the findings plausible/legitimate?) and ‘utility tests’ (are proposed solutions feasible in our context?) [140]. Consequently, local data is often most compelling, and contextualisation is needed for the findings to have leverage in the discursive and rhetorical processes that characterise policy-making [5, 141]. This suggests it may be unhelpful for interventions to focus solely on access to untailored systematic reviews and syntheses. Enhancing research for policy purposes involves trade-offs – increasing one attribute (relevance, credibility or accessibility) is likely to be at the expense of another. For example, presenting research findings clearly can enhance accessibility and relevance, but may also neglect important complexities, which decreases credibility. Therefore, solutions may be most effective when tailored on a case-by-case basis [142, 143].

Most of the interventions that attempted to increase access appeared to conceptualise access as necessary but insufficient for effective use of research, hence their parallel attempts to address individual, interpersonal and organisational capabilities. They recognise that research use is an intensely social and relational process [12], and that to increase it we have to understand and work with supporting factors such as organisational culture, professional behaviours, local circumstances and different intervention facilitation approaches [57, 144–147].

### Skills

Training workshops – the primary intervention for individual capacity-building – appear to provide a useful starting point providing they are well-tailored (resulting in relevant and appropriately pitched content) and facilitate active input from participants. Workshops are generally well received with high levels of self-reported improvement in understanding, but as a stand-alone intervention method they seem unlikely to result in substantial practice change. Uneke et al. praise the merits of one-off workshops [132], but follow-ups of RCTs find workshops alone to be costly and largely ineffective [85], indeed without support structures and systems even the best training will not be translated and sustained in practice [25, 102, 104]. The trade-off between intervention intensity and attendance by busy policy-makers, especially those at higher levels of seniority who also have a role in modelling and championing change, remains problematic.

The use of mentored practice seems to address some of these concerns, and is supported in the wider literature. The hypothesis that mentoring develops knowledge, skills and confidence has been tested in multiple studies with success where the mentor is appropriate and the mentor/mentee relationship is sound [148, 149]. Wider benefits include connecting mentees’ to communities of practice, and inspiring them to become mentors themselves [150]. Shroff et al. [103] suggest that, in policy agencies, this requires that the mentor has local knowledge and applied policy expertise. Others note difficulties in identifying mentors and matching them with mentees, and in sufficiently freeing up mentors’ time [54, 151].

Combining training and mentoring with performance goals and assessment (as three of the reviewed studies did) may offer the best option for embedding skills. For example, in their multi-country study Pappaioanou et al. conclude that “*without supportive follow-up and supervised application of skills, participants frequently continued to use the same work practices that they had used before they attended the training*” ([102], p. 1935). A recent meta-analysis found that goal-focused mentoring (otherwise known as coaching), even when short-term, improved individual, skills-based and affective outcomes [152]. Mentoring may offer greater support to staff who are less engaged in the workforce, so policy-makers who are new employees and/or especially lack confidence in using research skills may benefit most [151]. The terminology in this area is muddled so it is important to consider the specific tactics and goals of the intervention rather than relying on terms such as knowledge brokering, coaching or mentoring to define them.

### Systems

Knowledge utilisation is intimately linked to organisational structure and systems [38], so it is not surprising that these appear to play a key role in supporting individual efforts to access and use research. However, they must be fit-for-purpose, attuned to real practice needs, able to accommodate local adaptations and provide a clear benefit. Those developing and implementing such systems cannot afford to neglect the complex human dynamics within which they must work; consequently, participatory development of systems interventions may offer the best chance of success.

The outcomes of research-focused recruitment and performance management were not generally available in these studies, partly because their effects are often hard to disentangle from other strategies; however, they promise proximal and distal benefits. In-house research experts such as knowledge brokers may be more able to provide highly relevant, applicable, accessible and timely findings, but can also help to build wider capacity by supporting their colleagues’ skills development and contribute to a more research-orientated organisational culture. Evaluations of knowledge brokering in Scottish government departments and in Canadian healthcare organisations show a positive impact on research use [153, 154], and their use has been found to strengthen the clarity of research commissioned by policy-makers [155]. Our review found that the use of onsite knowledge brokers had mixed results, possibly because of the time needed to build productive working relationships with policy staff [133]. Thus, longer-term use may be most beneficial. Findings concur with descriptive and other empirical studies about the importance of knowledge brokers’ interpersonal skills and credibility [156–158].

### Interaction

There is little doubt that interaction between policy-makers and researchers – when it is positive and productive – tends to operate as a virtuous circle that increases trust and confidence in the benefits of further dialogue, and builds the capacity of both parties to understand and work with the other. For example, strategies that modelled respect for policy-makers as ‘knowers’ as well as ‘doers’ may have increased engagement, e.g. having senior policy-makers co-facilitate deliberative forums [132]. Mutual respect and commitment seemed to be crucial mechanisms, suggesting that those selected to take part in these initiatives should be carefully selected where possible, and that enthusiastic but sensitive facilitation might be helpful in the early stages. Results also suggest that reflexivity and continual adjustment may be crucial in dealing with the inevitable challenges of collaboration. There are tools available to help parties prepare for partnership work (e.g. [159]), and to monitor and evaluate its functioning [61, 62].

The extent to which interaction translates into research-informed policy-making is less certain. Neither increased understanding or collaborative outputs necessarily influence policy decision-making; however, where sound relationships are formed, they do appear to support the ‘social life’ of research, helping findings and ideas from research move into, within and between networks [160–163]. Empirical studies repeatedly find that professionals, including policy-makers, are more likely to seek and use research obtained via trusted interpersonal channels rather than from formal sources [38, 164, 165]. Interaction can build relationships that enable researchers to operate within this sphere of trust and familiarity [60, 165].

Despite disappointing outcomes in three of the five collaboration-focused studies, co-production remains a worthy goal, particularly in the light of a recent review that found an association between clinician involvement in research and improved healthcare performance [166]. The sticking point appears to be the capacity of individuals and organisations to facilitate genuine collaboration in which roles and tasks, resources and outputs are negotiated, and leadership is distributed across boundaries, resulting in shared expectations and mutually satisfying returns on investment. Early robust dialogue and fair but firm governance arrangements, underpinned by institutional support, seem to play an important role. The extent to which these policy-makers experienced a sense of ownership in the research process is likely to have been just as vital. As Zimmerman argues [167], ownership and buy-in are opposite concepts – ownership means collaborative development of ideas, decision-making and action, whereas buy-in means agreeing to someone else’s proposal. For example, in Kothari et al.’s [91] intervention the policy-makers’ involvement seems to have been limited to articulating the research questions and commenting on draft versions of the report. This consultative role places them closer to ‘endorsers’ than ‘co-researchers’ in the spectrum of co-production [65]. As Senge [168] puts it, people feel ownership of a shared vision not when they are playing according to the rules of the game, but when they feel responsible for the game. These findings align with other studies, including a systematic review which concluded that knowledge exchange in policy-making depends on the establishment of a viable cost-sharing equilibrium and institutionalised communication networks [43]. Evaluations of the CLARHC partnerships concur and draw attention to the benefits of leveraging existing relationships when starting partnerships [30, 169, 170].

### Key considerations in intervention design

The lack of detail about needs/situation analysis and how it was used to tailor interventions makes it hard to draw conclusions about the mechanisms that were or not triggered. However, others argue strongly that generalised capacity-building interventions are seldom successful; rather, they should be designed in response to accurate analysis of existing capacity and concerns, research needs and local conditions, derived from consultation or – better still – collaboration with potential participants [171]. This is supported by calls more generally for collaborative needs assessment as a precursor to local tailoring of interventions [172, 173], as this is likely to identify goals that are locally meaningful, make implementation plans more actionable, actively engage participants in translating data, and increase their investment in outcomes [174, 175]. Understanding existing capacity is also vital for tapping into local practice strengths [70]. As Trostle et al. argue, “*capacity can often be increased more effectively by reinforcing existing structures than by building new ones*” ([176], p. 63).

Findings emphasised the power of organisational culture to shape receptivity to intervention ideas and resources; this suggests that tailoring should take account of these dynamics. Where the existing culture is not perceived by staff to value research, the intervention may need to target values, beliefs and leadership prior to (or in parallel with) the other strategies. Attention to an organisation’s history and self-narrative is essential for crafting strategies that will resonate in current circumstances [100, 171].

Two-thirds of the studies used multiple strategies and targeted multiple domains of capacity. This is unsurprising given that supports and constraints in one area of capacity are known to influence capacity in other areas [39, 50]. It is outside the scope of this review to discuss the relative merits of single- versus multi-component interventions – plus others have dealt with this effectively elsewhere [177] – but it does seem that the degree to which intervention strategies are selected, tailored and implemented for local needs and practices may be more important than how many strategies are used, or in what combination [178]. Further, treating capacity-building as a participative endeavour is most likely to generate relevant, locally owned strategies [40, 176, 179]. We note that a 2011 meta-review of interventions designed to increase the use of research in clinical practice found that systematic reviews of multifaceted interventions reported greater effect sizes than single component interventions such as audit and feedback [180]. However, the extent to which these interventions were tailored for local needs, or developed collaboratively, is not reported.

The cumulative findings of these studies are a reminder that interventions are complex systems thrust into complex systems [77]. Research utilisation interventions, like other interventions, succeed or fail via their interaction with context – people, places, circumstances and processes will determine what works where, and for whom. Thus, the ‘best’ strategies for effecting change are those that are most fit-for-purpose at the local level [181]. We are warned of the considerable challenges that attempts to build capacity present. For example, that, “*… capacity-building is a risky, messy business, with unpredictable and unquantifiable outcomes, uncertain methodologies, contested objectives, many unintended consequences, little credit to its champions and long time lags*” ([56], p. 2). Nevertheless, this review shows that there are successes, and informative failures, so we can continue to develop our understanding of how to foster capacity in further interventions.

### Implications for future interventions

We note some areas that might be addressed fruitfully in further research-use capacity-building interventions in policy agencies:

- **Understanding research use in context:** Several studies in our review concluded that they had insufficient understanding of the local practices and contexts that were being addressed. This aligns with wider arguments that we continue to have a limited understanding of how policy-makers engage with research ideas and integrate them with other forms of evidence [26], which affects how we conceive of and design interventions, and interpret findings. Designing interventions that are “*close to practice*” [182] in terms of fit with local research needs and context seems to be essential, but we may also require further investigation of the ‘irrational’ (aka differently rational [6]) and non-linear uses of research that dominate the use of research in policy more generally. One of the reviewers of this paper pointed out that the consistency of contextual factors between our findings and other studies (which we attribute here to enduring contextual regularities in policy-makers’ use of research) may, in fact, be an artefact of an enduring research paradigm. The reviewer states, “*it could also be because much of the research into evidence use is conducted from identical premises (more research should be used) and using identical methods (surveys or interviews asking why more research isn't used).*” This is an important reminder of the need to ensure that the theories, models and methods we use in investigating research use are sensitive to real world policy practices and contexts rather than perpetuating a ‘barriers and enablers’ framework that risks masking complex interactions, identities and processes [183–185].
- **Researchers**’ **capacity:** Research-informed policy-making requires that researchers have the skills to produce policyrelevant research, present findings accessibly, and work productively with policy-makers; but these skills are often lacking [6]. Six of the reviewed studies attempted to build some aspect of researchers’ capacity in conjunction with that of policy-makers [62, 127, 131, 132, 137, 138]; however, a cursory scan of the literature suggests that capacity-building for researchers in this field is less developed than for policy-makers, with very few intervention trials; the onus remains on policy-makers. This appears to be a gap that would benefit from further attention. It would likely require that policy-makers are involved in designing the content of such interventions. Many of the mechanisms suggested in our analysis are likely to be relevant.
- **Leadership:** Findings reinforced the role of impassioned and strategic leadership as a crucial driver of organisational change (e.g. [103, 104, 128, 133]), including leadership by high profile external experts in the wider policy environment [102]. However, there seemed to be few attempts to target or harness internal leadership in intervention activities. The pivotal role of organisational leaders, champions and opinion leaders in driving change is well established both in practice settings [57, 186–188] and within policy agencies [18, 44, 50, 51]; but we know little about how leadership dynamics within hierarchical and procedure-focused policy agencies function and effect change in relation to research utilisation capacity-building. Recent arguments about the strengths of distributed or collective leadership for knowledge mobilisation, including cross-sector partnerships, suggest that our conceptualisation of leadership may need to expand [32, 100, 170]. This area could benefit from further investigation.
- **Audit and feedback:** With the exception of Peirson et al. [104], none of the studies reported using organisational-level progress feedback as a strategy, and none used audit and feedback – a process that gathers information about current practice and presents it to participants to facilitate learning, develop goals, create motivation for change and focus attention on change tasks [189]. Audit and feedback is well-established as a catalyst for professional practice change [189], including the uptake and use of research [144, 190]. There is mixed evidence for its effectiveness, but a recent systematic review found that it generally leads to small yet potentially important improvements in professional practice [191], and it may be more successful than change techniques such as persuasion [192]. It seems a potentially valuable strategy within research utilisation interventions, particularly in the light of systems-influenced implementation frameworks that emphasise the need to establish performance feedback loops in organisational change processes [10, 100, 108].
- **Commissioning research syntheses:** Findings of the two studies that looked at commissioned research syntheses suggest that the value policy-makers attribute to syntheses is affected by the commissioning process and/or their involvement in the conduct of the review [29, 91]. A contribution mapping review of 30 studies found that research was most likely to be used when it was initiated and conducted by people who were in a position to use the results in their own work [193]. However, an evaluation of health policy-makers’ use of a briefing service provided by academics found that access to the service did not improve the policy-makers’ capacity, nor their uptake and use of research [194]. What critical factors are at play in these scenarios? We would benefit from greater understanding of how commissioning models can best support policy-makers’ capacity development and use of research, including the contribution that researchers and knowledge brokers can make.
- **Sustainability:** The concept of capacity-building is linked to that of sustainability [70], but sustainability itself was seldom mentioned in the reviewed studies. As bureaucracies, policy organisations are characterised by their adherence to protocol, but there appeared to be few attempts to embed strategies within existing work systems (with some notable exceptions, e.g. [104, 134]). The need for continuous active participation in knowledge mobilisation practices [70] was evident in few studies. Several tried to embed new knowledge and skills in practice (e.g. via mentored assessment), but this targets individual knowledge rather than organisationally owned processes, which is an important consideration in organisations known for their high turnover [40]. Sustainability may depend on different mechanisms from those posited here. For example, self-efficacy may be critical for initiating new patterns of behaviour, but have a limited impact on the decision to maintain that behaviour over time [195]. Greater consideration of organisational learning and the use of measures to prevent capacity initiatives from being ‘washed out’ [196] may be required. Longer-term evaluation would help, but organisational change, like relationship building, is a lengthy and evolving process, often taking years to reach intended goals [62, 104].
- **Underpinning assumptions about research**-**informed policy-making:** Despite the lack of clear theoretical drivers in most studies, the conceptual basis of attempts to address research use in policy-making seems to be maturing – rational linear models of research are being supplanted by ideas from political science, organisational change, systems thinking and other bodies of work that disrupt the evidence-based policy ideal. The field is also making use of opportunities to evaluate capacity-building endeavours that are initiated outside of academia, using creative methods to learn from complex real world projects and refusing to be cowed by the entanglement of change strategies, process indicators and outcomes. However, there is still evidence of “*theoretical naivety*” as described by Oliver et al. [26]; for example, focusing on research as exemplary evidence rather than on policy-makers’ use of diverse information and ideas within which research must function; the belief that a reconfiguration of barriers and enablers to accessing research would lead to greater impact; and a general lack of understanding about policy processes. Oliver et al. [26] provide advice about the direction that future research can take to address these issues.

### Strengths and limitations

This paper contributes to our understanding of research utilisation interventions in policy agencies by providing an overview of 22 studies, including their change strategies and outcomes, the contextual factors that mediated these effects, and the theoretical perspectives that underpinned them. It also tentatively identifies the mechanisms that can best explain how the intervention strategies achieved their effects, or why they did not. This is an important first step in developing a more theoretically grounded approach to the design and evaluation of such interventions.

The paper may best be described as a realist-informed scoping review [197]. Unlike an orthodox realist review, it was conducted to inform our own program of research so it was not negotiated with external stakeholders, we did not conduct extensive theory-focused searches, and the analysis was exploratory and inductive rather than an interrogation of program theory [75]. We took an inclusive approach to study design and quality and, given our aim of identifying causal mechanisms, focused on identifying explanations of why an intervention was more or less successful rather than on quantitative findings [198]. The realist perspective contributed importantly to this process by enabling us to identify tentative constructs that may be used to inform the development, implementation and evaluation of subsequent research-to-policy capacity-building trials.

The findings are strengthened by independent analyses and critique of draft SCMO configurations, but our limited timeframe prevented the use of strategies that might have strengthened the review’s rigour further such as more comprehensive searching – this was an exploratory trawl of the literature rather than an exhaustive search – and contacting the authors of studies for missing information. The distinction between different participant groups and the extent to which the results for the various groups could be identified in the findings was not always clear. Including studies in which evaluations focused on processes and perceptions limits the identification of distal outcomes. The (mostly qualitative) data provided rich clues about contexts and possible mechanisms, but often did not include concrete information about capacity impacts or about any actual use of research in policy processes. Consequently, the findings should be seen as preliminary.

The identification of outcomes is further complicated by the entanglement of intervention strategies and outcomes. For example, improved infrastructure for accessing research, greater advocacy of research by organisational leaders, workforce development, and increased interaction between policy-makers and researchers can be seen as both intervention inputs and outputs – the means and the ends of capacity-building – depending on the focus of the intervention. As such, they tend to be described rather than evaluated. Many of the phenomena being investigated in these studies are complex and evolve over time; a reminder that capacity for using research in policy-making is a work in progress and will never be fully ‘built’.

Lack of shared evaluation frameworks across the studies means that we were not comparing like with like. A theory-driven approach in which we examined each study in relation to a middle range hypothesis could have produced more focused findings. We took an inductive approach in this first attempt, but believe that subsequent reviews would benefit from a theoretically based investigation. Our review might inform the development of causal hypotheses that further reviews could use as an investigative framework. The results tables (in which we present intervention strategies, contextual factors, hypothesised mechanisms and potential process effects/outcomes) reflect this exploratory approach. Not all of the elements are fully connected, lessening their explanatory potential [199]. We hope that future work will build on these lose connections to produce tighter configurations.

Lastly, mechanisms are “*squishy*” [200]. They change position in SCMO configurations, morphing into contexts and outcomes depending on the focus of the evaluation and level of analysis [31]. They can be differently aggregated, their categorisation is limited by vocabulary and interpretation [200], and their status as causal explanatory devices is uncertain; as Gerring argues, “*mechanisms might also be referred to as a theory, theoretical framework, or model, depending on one’s predilection*” ([200], p. 1503). Thus, the concepts we have called mechanisms, the level of granularity at which they are expressed, and the terms we use to describe them are all uncertain, and many would likely look quite different in the hands of another team. However, we believe that they offer a starting point for further testing and discussion. Typically, middle range theories develop gradually over time based on the accumulation of insights acquired through a series of studies [201]. This review is an early step.

## Conclusion

This review explores what intervention strategies have been trialled for building capacity to use research in policy-making, and tentatively posits possible mechanisms that might explain how those strategies functioned (or why they did not) in different contexts. The evidence is variable, especially because we included formative and process evaluations that focus more on process effects than measurable outcomes, but our findings suggest that tailored interactive workshops supported by goal-focused mentoring, and genuine collaboration, may be particularly promising strategies. Systems supports (e.g. infrastructure, governance arguments and workforce development) are likely to play a vital role, but it is very hard to disentangle their effects from other intervention strategies and systems flux. Many potential mechanisms were identified as well as some contextual factors that appeared to impact the functioning of virtually all intervention strategies. There were some gaps in the reviewed literature that could usefully be addressed in further research.

## Additional files

Additional file 1: Review characteristics and search strategy. (DOCX 38 kb)

Additional file 2: Overview of included studies. (PDF 389 kb)

## Abbreviations

SCMO: strategy + context + mechanism = outcome
