# Supplementary material for: What can we learn from interventions that aim to increase policy-makers’ capacity to use research? A realist scoping review
Source: Health Res Policy Syst. 2018 Apr 10;16:31. doi: 10.1186/s12961-018-0277-1 (PMC5892006; doi:10.1186/s12961-018-0277-1)
Supplement: Supplementary file 1 — Review characteristics and search strategy. (DOCX 38 kb) [file 12961_2018_277_MOESM1_ESM.docx]

# Additional file 1. Review characteristics and search strategies

**Table 1. Features of this review in relation to characteristics of scoping and rapid realist reviews**

| **Elements of a review** | **Characteristics of scoping reviews** [[1-4](#_ENREF_1)] | **Characteristics of (rapid) realist reviews** [[5-8](#_ENREF_5)] | **Characteristics of this review** |
| --- | --- | --- | --- |
| **Aims** | To map and summarise a body of literature, identify useful findings, and identify research gaps. Stakeholder consultation may be used to increase the usefulness of findings | To advance understanding of which interventions work for whom, in what circumstances, and how. Rapid reviews engage stakeholders in the process | Our aims correspond with both types of review, but no stakeholders were involved as the review was to inform our own program of work |
| **Research question** | A broad question to investigate what has been done in a field | A realist question that tackles the issue of what works for which groups in which circumstances | The research question is exploratory and reasonably broad, but with a realist focus on mechanisms |
| **Search strategy** | Inclusive (e.g. often incorporates grey literature), iterative, transparent but not necessarily replicable due to use of citation snowballing etc. | Same as for scoping reviews but guided by an initial program theory that is refined throughout the search. Often trans-disciplinary | Our search strategy corresponds with that of a scoping review. No overarching program theory or causal hypothesis was used to frame the search or analysis |
| **Additional information** | Optionally, authors may be contacted for supplementary information | Authors may be contacted for supplementary information, but this is unlikely when the review is rapid. Explanatory theory is sought | Authors were not contacted. Explanatory theory was sought, but not with the dedication associated with a high-quality realist review |
| **Quality appraisal** | Quality criteria are usually either low threshold or none are used. Critical interpretive synthesis (CIS), which shares some commonalities with scoping reviews, excluded only papers that are judged to be “fatally flawed” [[9](#_ENREF_9)] | Quality is assessed in relation to the review question: 1. Relevance – if it can contribute to theory building and/or testing; and 2. Rigour – if the method used to generate that data is credible and trustworthy | Our approach is closest to scoping reviews and CIS in that we prioritised relevance re the studies’ goals, strategies and participants rather than theory-building. Relevant studies were included providing they were not “fatally flawed” [[9](#_ENREF_9)] |
| **Data extraction and synthesis** | A descriptive analytical approach that includes process and theoretical information | Focuses on demi-regularities, middle range theories, context+mechanism=outcome patterns | We used realist methods, following the six-stage approach described by Best et al. [[10](#_ENREF_10)] |
| **Presentation of findings** | Depends on aims of review and types of studies included. May be tabular, narrative or both. | Incorporates context+mechanism=outcome patterns in tables, figures and/or propositions. Focus on theory-building and testing | Key findings are presented in context+mechanism=outcome configurations |
| **Philosophical underpinnings** | Unspecified | Realist epistemology and ontology, including assumptions about causality | Our philosophical stance is realist |

**Table 2. Database search strategies**

| **Search strategies and rationale** | **PAIS** (formally Public Affairs Information Service) | **Web of Science (WoS)** |
| --- | --- | --- |
| **Syntax used** | ALL fields: (intervention OR program*) AND (research OR evidence OR knowledge OR data) AND (policy-makers OR "policy makers" OR policymakers OR "government department" OR (government AND decision-makers OR "decision makers") OR "policy agency" OR "policy organi?ation") AND (capacity OR capability OR "professional development" OR skills OR training OR collaborat* OR partner*) | TOPIC: (intervention OR program*) AND (research OR evidence OR knowledge OR data) AND policy-makers OR "policy makers" OR policymakers OR "government department" OR (government AND decision-makers OR "decision makers") OR "policy agency" OR "policy organi?ation") AND (capacity OR capability OR "professional development" OR skills OR training OR collaborat* OR partner*) |
| **Additional filters** | Language: English  Source type: Reports, Scholarly Journals  Date range: 2001 to 2016 | Language: English  Documents: Articles  Date range: 2001-2016  Indexes: SCI-EXPANDED, SSCI, A&HCI, CPCI-S, CPCI-SSH, ESCI, CCR-EXPANDED, IC  Refined by: HEALTH POLICY SERVICES |
| **Articles returned** | 215 | 255 |
| **Rationale for selecting this database** | PAIS is considered to be the world’s largest database for public policy-related peer-review and grey literature [[11](#_ENREF_11)]. It was selected because of its breadth in content, including government and NGO reports, which supplemented the more academic databases used by the Moore and Campbell reviews. Also for its orientation to political science - a discipline that is strangely neglected in the research utilisation literature, even where it pertains to policymaking [[12](#_ENREF_12), [13](#_ENREF_13)]. Harrow [[14](#_ENREF_14)] describes the concept of capacity building as *“theoretically homeless”* but best served by the literature on public administration, stewardship and community development—all of which are included in PAIS. | WoS was selected because it is the world’s largest collection of research across the sciences, social sciences and humanities. However, we only searched within one category—health policy services—due to the unmanageable number of returns without that filter (2311). WoS also provides excellent citation searching which was used for snowball searching. |

## References

1. Colquhoun H: *Current best practices for the conduct of scoping reviews*. In *Impactful Biomedical Research: Achieving Quality and Transparency*. University of Toronto; 2016.

2. Arksey H, O'Malley L: Scoping studies: towards a methodological framework. *International Journal of Social Research Methodology* 2005, 8:19-32.

3. Dijkers M: What is a scoping review? *KT update: Center on Knowledge Translation for Disability and Rehabilitation Research* 2015, 4.

4. Colquhoun HL, Levac D, O'Brien KK, Straus S, Tricco AC, Perrier L, Kastner M, Moher D: Scoping reviews: time for clarity in definition, methods, and reporting. *Journal of Clinical Epidemiology* 2014, 67:1291-1294.

5. Saul JE, Willis CD, Bitz J, Best A: A time-responsive tool for informing policy making: rapid realist review. *Implementation Science* 2013, 8:103.

6. The RAMESES Project, *Quality standards for realist sysnthesis (for researchers and peer-reviewers)* 2014, The Rameses Project. Available from: [www.ramesesproject.org](http://www.ramesesproject.org). Accessed: 27 Feb 2017.

7. Wong G, Greenhalgh T, Westhorp G, Buckingham J, Pawson R: RAMESES publication standards: realist syntheses. *BMC Medicine* 2013, 11:21.

8. Wong G, Westhorp G, Pawson R, Greenhalgh T, Realist synthesis: RAMESES training materials, 2013, RAMESES 1 Project: Oxford. Available from: [www.ramesesproject.org/media/Realist_reviews_training_materials.pdf](http://www.ramesesproject.org/media/Realist_reviews_training_materials.pdf).

9. Dixon-Woods M, Cavers D, Agarwal S, Annandale E, Arthur A, Harvey J, Hsu R, Katbamna S, Olsen R, Smith L, et al: Conducting a critical interpretive synthesis of the literature on access to healthcare by vulnerable groups. *BMC Medical Research Methodology* 2006, 6:35.

10. Best A, Greenhalgh T, Lewis S, Saul JE, Carroll S, Bitz J: Large-system transformation in health care: a realist review. *The Milbank Quarterly* 2012, 90:421-456.

11. ProQuest, *PAIS Index*. Available from: [www.proquest.com/products-services/pais-set-c.html](http://www.proquest.com/products-services/pais-set-c.html). Accessed: 25 Nov 2016.

12. Liverani M, Hawkins B, Parkhurst JO: Political and institutional influences on the use of evidence in public health policy. A systematic review. *PLoS ONE* 2013, 8:e77404.

13. de Leeuw E, Clavier C, Breton E: Health policy – why research it and how: health political science. *Health Research Policy and Systems* 2014, 12:55.

14. Harrow J: Capacity building as a public management goal - myth, magic or the main chance? *Public Management Review* 2001, 3:209-230.
