# Supplementary material for: What can we learn from interventions that aim to increase policy-makers’ capacity to use research? A realist scoping review
Source: Health Res Policy Syst. 2018 Apr 10;16:31. doi: 10.1186/s12961-018-0277-1 (PMC5892006; doi:10.1186/s12961-018-0277-1)
Supplement: Supplementary file 2 — Overview of included studies. (PDF 389 kb) [file 12961_2018_277_MOESM2_ESM.pdf]

Additional file 2. Overview of research utilisation capacity building intervention studies aimed at policymakers

Study reference	Study design, goals, intervention strategies and domain	Participants and setting	Evaluation methods	Outcomes of interest / measures[1]	Theories, models, frameworks &/or theses	Results
**Study 1.**<br><br>**Brennan et al. 2015** [1]	Intervention study[2] evaluating policymakers' perceptions of relevance and potential impact of a long-term Policy Liaison Initiative (PLI) aimed at supporting the use of Cochrane systematic reviews in policy work.<br><br>Intervention strategies included: a community of practice to increase awareness and support knowledge sharing, seminars, skills workshops, a tailored website and review summaries.<br><br>Domain: Access, Skills improvement and Interaction	Targeted participants: Policymakers at managerial and lower levels in the federal Department of Health.<br><br>Participants n=unknown<br><br>Country: Australia	Individual interviews with participants: n=10/38 managers (who were randomised and sent personalised invitations). Seven group interviews n=33/5000 staff across all levels of the Department (who were sent general invitations). Plus participation data.	Use and awareness of systematic reviews<br><br>Awareness and relevance of PLI<br><br>Individual-, unit- and organisation-level capability to assess, interpret and apply research<br><br>Links with researchers and other external experts	Referenced literature regarding the complexity of policymaking (e.g. [2, 3]), the need for accessible research, and the value of high quality systematic reviews as efficient decision-making aids (e.g. [4, 5]). The selection of study outcomes was informed by arguments that distal research use cannot be wholly attributed to capacity-building (e.g. [6]). Data analysis was guided by the theoretical domains framework [7] and built on themes in previous studies (e.g. [8]).	Despite >565 occasions of attendance at forums and 294 members, most interviewees were not aware of PLI. They used reviews/syntheses but most did not distinguish between these and systematic reviews. Some did not understand the scope of systematic reviews. Access was impeded for those who found the Cochrane library hard to navigate. Links with researchers bolstered capacity to access and use research. Policy-relevance, applicability and accessibility were key needs. Managers were more confident than general staff that the Department had the skills to acquire, assess and interpret research.

[1] Not all of the outcomes of interest / measures are explicitly stated in the articles. Many are inferred from the descriptions of data collection and results.

[2] Three terms are used to describe the study design: *Experimental* = some form of randomisation and control groups were used, *Intervention* = the research team provided the intervention and evaluated it, but not using experimental methods, and *Observational* = the intervention or initiative being evaluated was not designed as part of a research study.

Study reference	Study design, goals, intervention strategies and domain	Participants and setting	Evaluation methods	Outcomes of interest / measures	Theories, models, frameworks &/or theses	Results
**Study 2.**<br><br>**Brownson et al 2011** [9]	Experimental study to identify the factors that influence whether state policymakers would find evidence briefs[3] about mammography screening understandable, credible and useful.<br><br>States were stratified and participants randomised to four groups, each receiving one type of brief: 1. data-focused brief with state-level data, 2. data-focused with local-level data, 3. story-focused with state-level data, 4. story-focused with local-level data.<br><br>Domain: Access	Three groups of state-level policymakers from six states: state legislators (elected officials), legislative staff (those serving the legislators), and health executive branch administrators (civil servants).<br><br>Participants n=840<br><br>Country: USA	Questionnaire-based study. Post-intervention survey responses n=291, an overall response rate of 35%, but a 47% response rate from executive branch administrators (the group of interest in this review)	Whether the brief was understandable, credible, likely to be used, and likely to be shared	Described the contradictory and overwhelming volume of information policymakers receive, and their preference for concise, relevant syntheses (e.g. [10, 11]). Noted the power of narrative in policy communication and the composition of effective policy briefs (e.g. [12]). Stories were crafted as per Kreuter et al.'s [13] framework. Data collection and analysis referenced personal and professional influences on policymakers' info engagement [e.g. 14, 15].	All recipients found the briefs understandable and credible.<br>67% of executive policymakers reported the briefs contained an appropriate amount of information, but 20% wanted more. This group were more likely to use and to share data-focused than story-focused briefs. This was the same for legislators but not staffers who were most likely to use story-focused briefs. Participants favoured state-level rather than local data, but they all operated at state level so a regional policymaker cohort may have responded differently.
**Study 3.**<br><br>**Campbell et al. 2011** [16]	Observational evaluation of policymakers' satisfaction with the process and outcomes of Evidence Check, a program that helps policymakers commission high-quality rapid reviews of research in 6-8 weeks.<br><br>An Evidence Check involves: policymakers completing a commissioning tool, a knowledge brokering session to clarify needs, agreement on the review proposal, selection of suitable researchers, management and delivery of the review.<br><br>Domain: Access	Policymakers in state government who commissioned Evidence Checks during 2007-2008<br><br>Participants n=>30<br><br>Country: Australia	Interviews with eight policymakers who had commissioned Evidence Check reviews.<br><br>Independent researchers assessed the accuracy of six commissioned rapid reviews which were randomly selected.	Satisfaction with the knowledge brokering process<br><br>Satisfaction with agreed review questions and parameters<br><br>Relevance and policy impacts of the review product<br><br>Relevance and accuracy of reviews	Like study 1 (above), barriers to research use and the value of concise syntheses were identified; but the emphasis here was on the limitations of formal systematic reviews [e.g. 17]; policymakers' need for timely, accessible and applicable answers to specific questions; the benefit of linkage with researchers; and the use of knowledge brokers as expert boundary spanners who can facilitate communication and enable the production of better-targeted syntheses (e.g. [18-21]).	Participants reported high levels of satisfaction with the knowledge brokering process and the reviews produced. Knowledge brokering helped to: refine research questions, shape project parameters (e.g. scope, budget, timeframe), and facilitate communication with researchers. The reviews were seen as useful with mostly indirect impacts, e.g. informing policy deliberations and identifying evidence gaps. Independent researchers assessed the reviews as accurately reflecting the current body of evidence.

[3] The terms *evidence brief* and *policy brief* are often used interchangeably in the literature. We use one term—evidence brief—for all studies, irrespective of the term used by each study's authors.

Study reference	Study design, goals, intervention strategies and domain	Participants and setting	Evaluation methods	Outcomes of interest / measures	Theories, models, frameworks &/or theses	Results
**Study 4.**<br><br>**Dagenais et al. 2013** [22]	Observational evaluation of a 'knowledge transfer' strategy aimed at improving the use of research results by policymakers and other stakeholders.<br><br>Intervention strategies included the production and dissemination of evidence briefs, workshops for sharing evidence and experiences, forums, research dissemination, advocacy documents and communications. Nearly 50 activities.<br><br>Domain: Access & Interaction	Policymakers from the national Ministry of Health and regions, funders, NGOs and health systems managers in two health districts<br><br>Participants n=*"hundreds of people"*<br><br>Country: Burkina Faso	Mixed methods case study. Baseline survey, participant interviews (n=38) and document review.	Extent to which aspects of the strategy were perceived as helpful<br><br>Extent to which research findings were used	The authors note the trend towards models of knowledge translation that emphasise complexity and policymaker-researcher interactions [23]. Seminal research use typologies are referenced (e.g. [24, 25]). A framework of categories that support research use is identified from the researchers' previous studies which guide the data analysis (although not cited, this framework has much in common with the diffusion of innovations framework developed by Greenhalgh et al. [26]).	Few participants read the research documents. Dissemination workshops had the greatest impact as they bypassed the need for skills in reading, appraising and interpreting research and did not contribute to information overload. However, workshop attendance was uneven. It was hard to get Ministry staff, especially those at senior levels, to participate in the intervention or evaluation and there were no discernible impacts at national level. Some regional policymakers were unaware of the research findings, but others used them instrumentally, conceptually and tactically.
**Study 5.**<br><br>**Dobbins et al. 2001** [27, 28] [4]	Intervention study that tested the extent to which health decision-makers used policy relevant systematic reviews that were provided by the research team.<br><br>24-month trial with five systematic reviews (on topics of current policy relevance) disseminated once.<br><br>Domain: Access	'Public health decision-makers' included clinicians, program directors and program managers in public health units. [5]<br><br>Participants n=unknown, but 41 public health units participated<br><br>Country: Canada	Cross-sectional telephone survey with 141 of 147 invited decision-makers (96% response rate), and a self-administered organisational demographic questionnaire was completed by 35 of the 41 public health units (85%).	Extent to which policymakers used the reviews in decision-making<br><br>What characteristics predicted use at the levels of the review, the individual policymaker, the organisation, and/or environment	This study was framed by diffusion of innovations theories (e.g. [29]). It drew links between research use in practice and policy in relation to the impact of: multiple forms of evidence, the power of personal attributes and experience, and the complex processes whereby new initiatives are adopted (e.g. [30-32]). An unpublished (and undescribed) framework guided the study. Survey instruments derived from previous studies, two of which combined concepts from multiple studies, mostly in nursing (e.g. [32-35]).	63% of respondents said they had used at least one systematic review to make a decision. Reviews were most useful for program justification and planning, but had little impact on evaluation decisions. Predictors of use were: organisational position (managers and directors were significantly more likely to use a review than clinicians), expecting to use a review in the future, perceptions that the reviews were easy to use and compensated for limited critical appraisal skills. Their impact was rated more highly in agencies with higher existing levels of support for research use.

[4] The two articles cited in relation to this study as complementary articles about the same study, so aspects of both are synthesised here
[5] Public health units are municipal-level agencies with legislative responsibility for research-informed program planning and evaluation (see Kothari et al. 2005)

Study reference	Study design, goals, intervention strategies and domain	Participants and setting	Evaluation methods	Outcomes of interest / measures	Theories, models, frameworks &/or theses	Results
**Study 6.**<br><br>**Dobbins et al. 2009** [36]<br><br>*(see also Traynor et al. 2014 – study 17 below)*	Experimental (randomised controlled) trial comparing: (a) access to an online registry of systematic reviews, or (b) access to the registry plus tailored weekly messages, or (c) access to the registry plus tailored messages *and* knowledge brokering.<br><br>Health departments were stratified and randomly allocated to the three groups. Over the 12-month trial there was ongoing access to the registries. Tailored messages were sent weekly X7. Knowledge brokers communicated >once monthly, made a site visit of 1–2 days, and hosted workshops and webinars.<br><br>Domain: Access and systems improvement	Policymakers and program managers in regional and local public health departments.<br><br>Participants n=unknown, but 108 of 141 (77%) national health departments took part<br><br>Country: Canada	Telephone-administered surveys twice at baseline and once post-intervention. Questions had been previously used and tested for reliability and validity. Post-intervention data were collected from 88 of 108 (81.5%) participating public health departments.	Extent to which research was considered in a program-planning decision within the past 12 months<br><br>Use of research in policies and programs	Barriers to research use were identified, including time constraints, research availability, and policymakers' limited capacity to appraise and translate studies (e.g. [19, 33]). References the value of tailored and targeted messaging, and of knowledge brokers, to improve the use of systematic reviews (e.g. [37, 38]). The study is guided by a framework that integrates concepts from diffusion of innovations [29] about the stages of adoption of new initiatives, plus concepts from the authors own work about the characteristics that mediate the uptake of research.	In most policy areas, the intervention had no significant effect on evidence-informed decision making, with no significant difference between the three intervention groups in the extent to which research was used. In public health there was a significant between-group difference in research use only when access to both systematic reviews and tailored messages were combined [36]. Having access to an online registry of research appeared to have no impact at all. Knowledge brokering also appeared to be ineffective, but may trend toward a positive effect when organisational research culture is perceived as low.
**Study 7.**<br><br>**Dwan et al. 2015** [39]	Observational study of a facilitated engagement strategy that enables researchers to present contextualised findings to policymakers. Included validation of an evaluation instrument.<br><br>From 2008, 23 seminars (presentations with Q&A) and 13 roundtables (tailored interactive discussions) have been held. They have been facilitated by a knowledge broker since 2011.<br><br>Domain: Access and Interaction	Policymakers in the federal Department of Health<br><br>Participants n=1865(?)<br><br>Country: Australia	Post-forum questionnaires were completed, n=979, (52.5% response rate). Questions focused on effectiveness, relevance and receptivity.	Perceived effectiveness in broadening knowledge and stimulating thinking<br><br>Perceived relevance (work applicability)<br><br>Research receptivity (interest in and use of research in past 12 months)	The study is framed by literature focusing on the complexity of getting research into policy [40]. The goal is conceptualised as research mobilisation (rather than transfer or translation [41]), and linkage between researchers and policymakers [42] that counters the two-communities divide [43]. The need for tailored information, and the situated nature of research usefulness are emphasised [44, 45]. Forums were based on exchanges in previous studies (e.g. [46])	Participants indicated that the forums had broadened their knowledge and stimulated thinking. Over ¾ indicated the forums' content was directly applicable to their work and they may be able to use it. The content of roundtables was more applicable than seminars, but was no more effective in stimulating thinking and/or broadening participants' knowledge. International speakers were rated as especially effective. Nearly 90% had used research in the past 12 months and said they would use it more if it were easily available.

Study reference	Study design, goals, intervention strategies and domain	Participants and setting	Evaluation methods	Outcomes of interest / measures	Theories, models, frameworks &/or theses	Results
**Study 8.**<br><br>**Hawkes et al. 2016** [47]	Observational study of interventions to enhance the capacity of policymakers, policy organisations and wider policy influencers.<br><br>Situation analysis was used to identify capacity needs and inform intervention design. Strategies included: skills workshops, seminars, cross-sector retreats, research access infrastructure improvements (including a government-run portal for research syntheses), incorporation of research-policy topics in post-graduate health training, and establishing a government committee for commissioning reviews and advice.<br><br>Domain: Access, Skills improvement, Systems improvement, Interaction	Policymakers in government health departments, parliamentarians, senior health care managers and practitioners – varied according to country.<br><br>Participants n=unknown<br><br>Countries: Bangladesh, Gambia, India and Nigeria	Implementing sites conducted their own evaluation. Methods included: stakeholder interviews (Bangladesh, Nigeria), pre/post quantitative surveys of changes in knowledge, attitudes and practice (India, Nigeria), and document analysis of pre/post frequencies of references to research in Parliamentary discussions (Gambia). These evaluations were synthesised by an independent evaluator.	Extent to which the tailored intervention strategies addressed the capacity needs (individual, organisational and institutional) that were identified locally in each setting	The interplay of evidence and politics is noted [48, 49], and the capabilities required to use research effectively in this complex environment [50], which are often lacking in LMIC [51]. Capacity is conceptualised at multiple levels as per the UK Department of International Development [52]. Institutional capacity is regarded as especially critical for sustained research-informed policymaking. The authors cite Ward et al. [53] on the role of interaction as an explanatory feature in research transfer models, and frame the results using categories of capacity described by Moore et al. [54].	Skills workshops were well-received and generally well attended, and were successful in building individual capacity to access, understand and use research/data. Interactions and provision of policy-relevant summaries led to improvements in researcher-policymaker relationships. Organisational capacity to access research was strengthened via infrastructure supports (e.g. IT resources) and newly established interactive forums. The impacts on policymaking, however, were intangible. Institutional capacity was seldom addressed and the authors conclude that more needs to be done in this sphere. Lack of shared evaluation frameworks hindered the study.

Study reference	Study design, goals, intervention strategies and domain	Participants and setting	Evaluation methods	Outcomes of interest / measures	Theories, models, frameworks &/or theses	Results
**Study 9.**<br><br>**Hoeijmakers et al. 2013** [55]	Observational study of the effects of Academic Collaborative Centres (ACCs): partnerships between government, public health services and universities to support knowledge mobilisation.<br>Activities included jointly creating a program theory, collaborative research projects, education forums, and shared data infrastructure.<br>Domain: Access, Skills improvement, Systems improvement and Interaction	Policymakers and practitioners in municipal departments and public health services, and researchers in public health<br><br>Participants n=unknown<br><br>Country: the Netherlands	Three-year-long developmental evaluation using mixed methods: focus groups, interviews, network analysis and case studies.	Extent of outputs and outcomes as defined in program theory, e.g. collaboration on research and grants, structure for knowledge exchange, implementation capacity, research uptake and appraisal skills, new publications.	ACCs were conceptualised as boundary organisations [56] intended to support cross-sector collaboration that would, in turn, foster Research→Policy [57]. The study's program theory (which was programmatic rather than theoretical) guided data collection and analysis. Findings build on previous ACC [58] including a study that took an interpretive hermeneutic approach [59].	The ACC provided a platform for dialogue and interaction, but project collaborations did not extend into enduring partnerships. Most committees functioned well but thematic groups were less successful due to lack of support from managers. Overall, policymakers were less involved than researchers and practitioners. New research proposals were written but non-researcher involvement was limited and traditional research designs were used. The number of projects and participants increased over time, but the structure and density of networks was unchanged.
**Study 10.**<br><br>**Kothari et al. 2005** [60]	Experimental case controlled study that tested whether policymakers were more likely to use a research report if they were involved in its production.<br>12-month trial with ongoing feedback and one presentation for the three 'involved' units. Both they and the three comparison units received a copy of the final report.<br>Domain: Access and Interaction	Teams of general staff and managers in public health units<br><br>Participants n=unknown. Three teams received the intervention and three teams were selected as controls.<br><br>Country: Canada	Comparative multiple-case study design using group interviews with participants, individual telephone interviews with directors, and document review	Extent to which participants:<br>1. Received reports<br>2. *"Processed"* the reports (including if they assessed their merit and validity)<br>3. Applied the report i.e. used it conceptually or instrumentally	The authors hypothesise that formal policymaker-research linkage and exchange [42] will create shared agendas, solutions, practices, lexicon and goals that bridge the two-communities [43] and counter static research transfer models. The study was guided by a conceptual model of stages and types of research use ([61] and [49]).	Staff within units that were involved in the production of a research report were more likely to receive a report, and to understand it better and value it more, than units that were not involved. But actual use was not affected. Both involved and comparison units used the research findings to confirm that their program activities were consistent with evidence, and to compare their program performance relative to other units.

Study reference	Study design, goals, intervention strategies and domain	Participants and setting	Evaluation methods	Outcomes of interest / measures	Theories, models, frameworks &/or theses	Results
**Study 11.**<br><br>**Kothari et al. 2014** [62]	Observational study to determine the extent to which the PreVAiL (Preventing Violence Across the Lifespan) research network built effective partnerships among network members. PreVAiL is an international interdisciplinary public health network comprising researchers and policy/advocacy partners.<br><br>Strategies included: collective (Delphi process) research priority-setting, funded team meetings, seed grants for collaborative research and dissemination, and capacity development for early career researchers.<br><br>Domain: Interaction	Researchers (including trainees) and knowledge users (including policymakers in different parts of public health such as justice and child welfare)<br><br>Participants n=60 + 15 trainees<br><br>Country: Canada, but with partners in US, UK, Asia, Europe and Australia	Partnership Indicators Questionnaire (PIQ) completed by 36 PreVAiL members (n=26 researchers and n=9 partners) with a 65% response rate.<br>19 semi-structured telephone interviews conducted two years after network became operational.	Partnership quality within the network: levels of partner involvement, quality of communication, perceived value of network<br><br>Initial impacts of the partnerships on the application of knowledge to policy and practice (instrumental, conceptual)	Gaps between knowledge and practice [23] are tied to disconnects between researchers and knowledge-users [43]. Collaboration in research development and dissemination is described and advocated for. [63-66]. The authors argue that, despite recent reviews [67, 68], collaboration remains a 'black box' and greater understanding of partnerships is needed [69]. The PreVAiL network was based on public health approaches to violence. Collaborative development of the questionnaire and indicators is described elsewhere [45]. Thematic data analysis was used. [70]	Participation rates varied from 11-79%. The network was seen as beneficial for individuals and organisations. 75% of PIQ respondents felt their contributions were valued. Partners used the network as a source of synthesised information, but tended to contact the same researchers. Some partners functioned as an 'information conduit' to their own organisation. There were collaborations in writing papers, grants and speaking at events, but desire for greater collaboration on grants, research proposals and advocacy. Most knowledge was used conceptually, but there were examples of instrumental use. Not all policymakers felt there was a common language between network members.

Study reference	Study design, goals, intervention strategies and domain	Participants and setting	Evaluation methods	Outcomes of interest / measures	Theories, models, frameworks &/or theses	Results
**Study 12.**<br><br>**Langlois et al. 2016** [71]<br><br>(This paper reports on two studies. We only include the second one here, 'Policy BUDDIES', as the first does not meet our inclusion criteria)	Intervention study of a pilot initiative designed to build the capacity of policymakers to demand and use systematic reviews.<br><br>Strategies included: baseline situational analysis, skills workshops, the allocation of researcher 'buddies' to work with policymakers on refining research questions for reviews, and an online support system for buddies.<br><br>Domain: Skills improvement and Interaction	Policymakers in provincial government involved in programs related to Millennium Development Goals, and their researcher 'buddies'<br><br>Participants n=unknown<br><br>Countries: Cameroon and South Africa	The realist mixed methods evaluation included document review (e.g. technical reports, policy documents and news media), in-depth interviews with policymakers and project staff, and a focus group with researcher buddies. A previous descriptive study explored policy contexts and research needs [72].	Identify any lessons learnt about the process and impact of the Policy BUDDIES strategy	Focuses on the need for demand-driven research [73], and the importance of organisational culture in fostering research use [74]. Intervention design was based on studies highlighting the centrality of partnerships approaches and trusting relationships alongside the need to ensure that used research is robust and valid e.g. [75]. Data collection drew on Walt and Gilson's policy analysis framework [76]	Buddying helped policymakers to value and use research evidence, but also built the capacity of researchers to understand policy needs and provide useful support. Buddies were perceived as more objective than other experts. Interactions were necessarily iterative and required equality and trust. Institutional support and incentives for using research were important barriers/facilitators to policymakers' involvement in generating and using evidence. Champions drove policymakers' ownership of the initiative.
**Study 13.**<br><br>**Pappaioanou et al. 2003** [77]	Intervention study that tested an intervention for strengthen the capacity of policy staff to collect, analyse, report and use epidemiological data.<br><br>Strategies included the implementation of country-specific health information and communication systems, extensive skills training tailored for the different participant groups and mentorship in applying those skills.<br><br>Domain: Access, Skills improvement, Systems improvement	Policy decision-makers, program managers, technical experts, and information specialists in Ministries of Health<br><br>Participants n=unknown<br><br>Countries: Bolivia, Cameroon, Mexico and the Philippines	Data availability and use was measured at baseline, midpoint and 1-year after completion. Indicators were matched to country situations and project designs. Additional methods included participant interviews and country case studies.	Outcome indicators were matched to country situations and project designs. Examples include proportions of participants who:<br><br>1. satisfied minimum skills requirements post-training<br><br>2. presented their work 3. showed improvement on test scores<br><br>4. made data-based decisions	Draws on research utilisation literature re the limitations of rational research use models, the influence of political context [78], and the need to involve users in systems design [79]. Aimed to reduce barriers including: the failure of researchers to produce quality, timely, inaccessible research and lack of participation in interpretation [80]; poor systems for accessing policy-relevant information; and the need for policymakers to understand and trust health data. [81]	All countries trained policy staff (a) to use data and (b) to train others to use it. Participants reported the training taught them how to work as part of a public health team, empowered them to use data to identify critical health community problems, helped them understand their local decision-making environment, and helped them set achievable outcome-oriented goals and formulate and implement plans to tackle them. Quantitative skills assessment data is not reported. The intervention was found to improve data-informed public health in all countries. Some country-specific impacts are identified.

Study reference	Study design, goals, intervention strategies and domain	Participants and setting	Evaluation methods	Outcomes of interest / measures	Theories, models, frameworks &/or theses	Results
**Study 14.**<br><br>**Peirson et al. 2012** [82]	Observational evaluation of the implementation and impacts of a strategic plan for using research in decision-making.<br><br>Strategies included: skills workshops; developing literature review methods and tools; forums for sharing knowledge; restructuring and expanding the library; creating and supplementing research-related positions; accessing external expertise; and commissioning literature reviews.<br><br>Domain: Access, Skills improvement, Systems improvement	Medical officers, library staff, directors, managers, supervisors, and research and policy analysts in a Public Health Unit.<br><br>Participants n=unknown<br><br>Country: Canada	Longitudinal qualitative case study using data from two sets of purposively sampled semi-structured interviews (n=6) and focus groups (n=27) with 70 members of the health unit, and review of 137 documents.	The evaluation attempted to identify:<br><br>1. How capacity change was attempted in the implementation of the strategic plan<br><br>2. Practices and resources needed to carry out research use tasks<br><br>3. Incorporation of research in decisions and documents<br><br>4. Any influencers on the above	The intervention was developed using strategies from outside healthcare identified in an earlier study based on the hypotheses that research-informed policymaking requires a culture of critical inquiry, staff capacity and tools for research use, and improved organisational knowledge management [83]. Collaborative data collection and analysis was informed by key texts in organisational change (e.g. [26, 29, 84, 85]), knowledge exchange [23] and implementation [86]).	Over two years, staff confidence and skills increased, their literature reviews became more rigorous, research skills were built into job descriptions and evident in the changed workforce, and there was significant investment in further development. Critical factors for building capacity were identified as: strong continuous leadership, clear vision, workforce and skills development, improved access to research, fiscal investments, use of technology, better knowledge management, effective communication, receptive organisational culture and use of change management techniques
**Study 15.**<br><br>**Rolle et al. 2011** [87]	Intervention evaluation of a year-long program to build the capacity of government decision-makers to use HIV data strategically.<br><br>The intervention comprised block weeks of training in: HIV interventions and situational analysis; descriptive and analytic epidemiology; HIV surveillance; and evaluation. Regional teams were mentored by researchers to complete a practical project that they presented for assessment.<br><br>Domain: Skills improvement	Government employees (surveillance officers, public health laboratory technician, and project managers) in HIV/AIDS Prevention and Control Department<br><br>Participants n=23<br><br>Country: Ethiopia	Per-module and whole-course surveys, and post intervention focus groups with trainees (n=15) and stakeholders (n=6). Participants who withdrew were followed up. Group presentations were assessed by an expert panel.	Rates of course completion<br><br>Trainees' regard for the course<br><br>Trainees' application of knowledge and skills learned during the course<br><br>The contribution of this course to increasing skilled public health capacity in Ethiopia	Training modules derived from the *Centers for Disease Control and Prevention* which has 50+ years' experience of running successful training based on a *"learning while doing"* philosophy to build critical reasoning skills [88]. An adapted evaluation model was used that sought to identify mechanisms that enable participants to use data in decision-making. The original model posits that training can change behaviour by influencing beliefs about the value of practices, and by increasing knowledge and skills, thereby enhancing self-efficacy. [89]	92% of participants felt the course met their expectations and all said it was relevant to their work. Self-reported skills improved: trainees could collect, analyse and interpret data effectively and use the findings, and carry out work tasks confidently. The expert panel judged that trainees had learned core skills in using data but needed to refine their analyses and correct some errors. Some trainees went on to train their colleagues. Retention increased in subsequent cohorts (from 65% to 87% & 92%) after program improvements, e.g. a shorter more intensive course and the addition of mentors from outside academia. Mentorship was hypothesised to be the critical mechanism of change. Stakeholders agreed the course contributed to skills capacity in Ethiopia.

Study reference	Study design, goals, intervention strategies and domain	Participants and setting	Evaluation methods	Outcomes of interest / measures	Theories, models, frameworks &/or theses	Results
**Study 16.**<br><br>**Shroff et al. 2015** [90]	Observational evaluation of five projects resulting from a WHO initiative to "catalyse" the use of health research in policy via push, pull and exchange activities.<br><br>Interventions were specified locally but could include: platforms to produce and communicate research; training programs; establishing data usage units within ministries of health; developing and using evidence briefs; and hosting policy dialogues or other forums for connecting researchers and policymakers.<br><br>Domain: Access, Skills improvement, Systems improvement, Interaction	Participants included national and regional policymakers, and managers of healthcare institutions<br><br>Participants n=unknown<br><br>Countries: Argentina, Bangladesh, Cameroon, Nigeria and Zambia.	Interviews (n=22), an evidence briefs survey (n=167, 63% response rate), a policy dialogues survey (n=140, 60% response rate), and outcome evaluation surveys (n=66 in Nigeria, n=48 in Zambia. Review of project technical reports. Not all data sources were available for all countries.	To understand why interventions in some settings were perceived by the key stakeholders to have made progress towards their goals, whereas others were perceived to have made little progress	Referenced literature re the complexity of policymaking, different models for mobilising research, and how research is used (e.g. [8, 91, 92]). Choice of intervention strategies (evidence briefs, research packaging, policy dialogues and reflective forums) was based on previous studies (e.g. [93-95]). Jacobson et al.'s [96] theory of knowledge translation guided the identification of variables across five domains: user group, issue under consideration, research attributes, researcher-user relationships, and dissemination strategies.	There was considerable variation in intervention activities and their intensity. In more successful projects the use of research was aided by a combination of: enthusiastic policymakers in research-orientated ministries; research topics that were policy priorities and also interested the researchers; the availability of reliable, easy-to-understand research; positive research/policy relationships; clear expected outcomes; thorough dissemination of findings; and strong project leadership. The use of multiple strategies targeting different domains was thought to be beneficial. The practice of establishing research centres in ministries is suggested.
**Study 17.**<br><br>**Traynor et al. 2014** [97] [6]	Observational, mostly qualitative study of the role of knowledge brokers (KBs) in policy agencies conducted across two primary studies: Dobbins et al. 2009 (included above) and the unpublished (?) case study of a subsequent trial.<br><br>In the first study KBs were used as one strategy within a multi-strand RCT. In the second 22-month trial, KBs offered tailored services including group training, tools, management consultation, and intensive mentoring.<br><br>Domain: Access, Skills improvement, Systems improvement	Policymakers in regional health departments<br><br>Participants n=unknown, but study 1 targeted policymakers in 30 health departments, and study 2 targeted policymakers in three health departments<br><br>Country: Canada	Study 1. See Dobbins et al. 2009 above. Study 2 included social network data, close-ended surveys, interviews (n=37), organisational documents and reflective journals. Thematic coding based on frequency and emphasis of themes across data sources and studies.	The impact of knowledge brokers in two intervention strategies<br><br>The attributes of effective knowledge brokers<br><br>The impact of organisational context on knowledge brokering activities	Knowledge brokers with requisite skills are hypothesised to use interpersonal contact to build bridges across the research/policy divide and facilitate the development and/or uptake of policy relevant research (e.g. [98-100]). Data collection and analysis was informed by principles from case study [101] and qualitative research [102, 103] texts. Findings build on previous studies to suggest that KBs enhance individual as well organisational and cross-organisational capacity.	KBs were found to enhance individuals' capacity by improving knowledge, skill and confidence in searching for, appraising and applying research. Ongoing personal support throughout projects was more helpful than training or tools. Organisations used KBs to initiate train-the-trainer KB functions and research-oriented internal policies. Effective KB attributes included expertise in research and health, and personal traits of approachability and patience. Staff felt they could admit needing help because the KBs were 'external'. Use of KBs improved as relationships grew. KBs may require organisational support to be most effective.

[6] This article examines two studies, one of which is included in this review (Dobbins et al. 2009). However, the second study has not been published elsewhere as far as we can tell, so the article was eligible for inclusion as a primary study.

Study reference	Study design, goals, intervention strategies and domain	Participants and setting	Evaluation methods	Outcomes of interest / measures	Theories, models, frameworks &/or theses	Results
**Study 18.**<br><br>**Uneke et al. 2012** [104]	Intervention study of a program aimed at improving participants' capacity to acquire, assess, adapt & apply research in policymaking, and enhancing research/policy partnership links.<br><br>The intervention comprised a one-day evidence-to-policy training forum. There were four lectures of 30 minutes given by senior academics followed by interactive sessions.<br><br>Domain: Skills improvement	Policymakers (directors, project/program managers, department heads in the regional Ministry of Health and uniformed services), other health decision-makers (e.g. directors of NGOs, hospital administrators) and researchers.<br><br>Participants n=104<br><br>Country: Nigeria	Pre- and post-forum self-rated questionnaires. Six focus groups (n=9-12 people in each) were used to identify barriers to research-informed policy development.	Attendance rates<br><br>Improvements in participants' knowledge compared to their pre-forum status<br><br>Improvements in participants' use of evidence compared to their pre-forum status	Identifies the gaps between research and policy [105] lack of health agenda ownership by policymakers in low- and middle-income countries (LMICs) [106] and the non-linear, non-rational process of policymaking [107]. The use and design of workshops was founded on work by Poulos et al. [108]. Statistical data analysis used methods developed for LMICs [109] and phenomenological analysis of focus group data followed Giorgi [110]	Attendance rate of 84%. Of the 81 participants 64% were policymakers. Pre-forum these participants supported the intervention goals, and believed that research could provide sound and relevant guidance for more effective, efficient and sustainable health systems. Post-forum, participants reported they had greater understanding of how to: access research and assess its policy relevance; synthesise and present research; transform research into policy; and amplify the impact of research in policy.
**Study 19.**<br><br>**Uneke et al. 2015a** [111]	Intervention study aimed at improving participants' capacity to develop evidence briefs. This study extends Uneke et al. 2012 (study 17 in this table). Here they expand on their previous use of a one-day training forum to include two mentored group meetings aimed at supporting policymakers to identify and investigate the evidence for potential policy options. Research synthesis were developed for each option and those assessed as having the strongest evidence base were included in evidence briefs.<br><br>Domain: Skills improvement	State-level health policymakers involved in the control of infectious diseases.<br><br>Forum participants n=43/50 Mentored participants n=unknown<br><br>Country: Nigeria	A pre/post survey was administered for training forum participants (n=38, but only 21 appeared to be government policymakers). The process and outputs of mentored group meetings are described	Changes in participants' perceived knowledge of forum topics (collaboration, evidence briefs, policy dialogues, research ethics, and the local health policy context)<br><br>Capacity to produce evidence briefs	Poor use of research in LMIC is noted, and the need to strengthen policymakers' capacity to use research, including through evidence briefs [5]. They recognise the politicisation of policymaking and the need to incorporate different stakeholder perspectives in policy options [112]. Intervention design draws on studies that emphasise the benefits of training workshops and mentoring [113]. The introduction of formal group mentoring was based on guidance by the Canadian Coalition for Global Health Research [114]	Results showed improvement in participants' capacity to use research effectively in the development of evidence briefs. The self-reported post-workshop percentage increase in mean knowledge and capacity across the 5 workshop topics ranged from 21% to 46%. Mentored groups successfully produced briefs with research-informed options. The authors conclude that policymakers' knowledge and capacity to develop evidence briefs can be enhanced via a one-day training workshop followed by an intensive mentorship program.

Study reference	Study design, goals, intervention strategies and domain	Participants and setting	Evaluation methods	Outcomes of interest / measures	Theories, models, frameworks &/or theses	Results
**Study 20.**<br><br>**Uneke et al. 2015b** [115]	Observational evaluation of a program aimed at building the capacity of a newly formed health policy advisory committee (HPAC) to promote research-informed policymaking and function as a knowledge translation platform. Strategies included: a one-day interactive workshop on evidence briefs/dialogues and use of SUPPORT tools, a three-month university-based training and mentorship program focusing on research use in health policy and systems, collaborative development of an evidence brief and hosting of a policy dialogue.<br><br>Domain: Skills improvement, Interaction	Directors from the regional Ministry of Health (MoH) and representatives from other government organisations, an NGO director, and senior researchers.<br><br>HPAC members n=18, including n=9 MoH directors and n=4 other policy representatives<br><br>Country: Nigeria	Interviews with HPAC members who were: 1. workshop participants (n=8), 2. training and mentorship program participants (n=8),3. and policy dialogue participants (n=8). Given that the HPAC had cross-sector membership, the number of policymakers within each sample is unknown.	Participants' post-training understanding of knowledge translation<br><br>Capacity to use research in practice<br><br>Quality of relationships between policymakers and researchers	The intervention is modelled on HPACs in other countries and premised on the assertion that regular interaction between policymakers and researchers can address the gaps between them [112, 116]. Follows Choi et al. [117] who suggest that collaboration can increase policymakers' capacity to apply a *"science lens"* to policymaking, and researchers' capacity to be *"policy sensitive"*. Aims for a systematic and transparent appraisal of research within policy processes [5]. The evaluation drew on qualitative methods within a case study approach. [101, 118]	Participants' reported: increased understanding of practical knowledge translation, including how to access and use research; markedly reduced distrust between policymakers and researchers; and greater ability to promote research-informed policymaking within the Ministry of Health. The evidence brief produced by the HPAC has been published [119] and is under consideration by the MoH. HPAC members called for performance measurements and institutional support to ensure continuation and independence. The authors note the need for continual training and interaction if HPAC productivity is to be sustained.
**Study 21.**<br><br>**Waqa et al. 2013** [120]	Observational process evaluation of a tailored intervention to build policymakers' capacity to produce evidence briefs.<br><br>Strategies included: analysing existing organisational capacity; mapping the policy environment; skills workshops; access to WHO Hinari program; knowledge brokering & mentored development of evidence briefs (284 meetings in total), guided by advisory groups. Strategies were designed in collaboration with senior staff in each organisation who also acted as project advisors and coordinators.<br><br>Domain: Access, Skills improvement, Systems improvement	Four government agencies and two NGOs assessed as having the potential to make or influence health policies across diverse population groups and settings.<br><br>Participants n=49 in 6 organisations (5-12 in each). Four of these were policy organisations<br><br>Country: Fiji	Intervention activities were recorded using a data collection proforma that captured information on: implementation processes and the scale, duration, reach and frequency of activities. Process dairies were kept by project team members.	Duration, frequency and type of interaction and/or activity between the knowledge brokering team and participants<br><br>Increase in perceived skills to acquire, assess, adapt and apply research<br><br>Application of skills in producing an evidence brief	The authors hypothesised that increased researcher-policymaker interactions, facilitated by knowledge brokers [36], promote research use in policymaking [8, 26]. Intervention strategies were informed by previous studies, including: the use of an advisory panel, gaining high level organisational buy-in via 'concept papers' [121], targeted skills development [82], supported development and presentation of evidence briefs [122], assessment of existing skills and support for using research [123]. Use of process diaries was based on Waters et al. [124]	55% of participants completed the 12-18-month intervention, 63% of these produced one or more briefs (n=20) and 5 organisations developed templates for constructing future briefs. The knowledge brokering team spent an average of 30 hours per participant. Organisations with higher levels of internal support for using research developed more briefs. The program's success was built on partnership with high-level policy staff in each organisation which were formalised and resulted in strong organisational commitment to the project, but it was undermined by high staff turnover.

Study reference	Study design, goals, intervention strategies and domain	Participants and setting	Evaluation methods	Outcomes of interest / measures	Theories, models, frameworks &/or theses	Results
**Study 22.**<br><br>**Wehrens et al. 2010** [125] [7]	Observational study of how interactions within a 'Small But Beautiful' research-policy-practice partnership project contributed to outcomes<br><br>Strategies used in these 3-month research projects included interactive rounds of problem clarification, collaborative research design and report discussions, culminating in user-focused presentations of findings. A supervisory group comprised public health services staff and researchers, but not policymakers.<br><br>(This project was conducted under the auspices of the study evaluated by Hoeijmakers et al. 2013 above).<br><br>Domain: Access and Interaction	Participants included policymakers, managers, and epidemiologists from the city council, regional health services and university staff<br><br>Participants n=unknown<br><br>Country: the Netherlands	Case study informed by interviews with participants (n=16), project document review and observation of project meetings and seminars.	The nature of relationships within the partnership<br><br>How the partnership structured and responded to research/policy expectations and interactions<br><br>How the above impacted on content emerging from the partnership	This study referenced the trend away from linear research transfer and towards researcher-policymaker interaction [8, 42, 126, 127]. The lack of knowledge about how interaction contributes to research use is noted [63, 126]. It questions the hypothesis that structural support for interaction is sufficient to facilitate meaningful communication and connection [128]. Information about the intervention design is not available in English. No literature is cited as informing the data collection or analysis.	Goals were undermined by differences between partners in views, values and expectations. In the first presentation, policymakers perceived the researchers as poor communicators who were too focused on methodology, and found the results inaccessible and lacking policy usefulness. Results were repackaged using scenarios to highlight policy relevance, and a carefully managed public forum was held. This was perceived as successful in presenting scientifically robust findings that were also accessible and applicable. Findings have influenced problem definition and agenda-setting, and paved the way for further research-policy collaborations.

[7] This study is based on the same initiative as that of Hoeijmakers et al. 2013 (study no. 9 above) but was conducted at a different time point by different researchers and focuses on one regional project. Hoeijmakers et al. 2013 evaluate the intervention at a national level, hance they are treated separately

References

1. Brennan, S.E., et al., Design and formative evaluation of the Policy Liaison Initiative: a long-term knowledge translation strategy to encourage and support the use of Cochrane systematic reviews for informing health policy. *Evidence & Policy*, 2016. 12(1): 25-52.
2. Dobrow, M.J., et al., The impact of context on evidence utilization: a framework for expert groups developing health policy recommendations. *Social Science & Medicine*, 2006. 63(7): 1811-1824.
3. Dobrow, M.J., V. Goel, and R.E. Upshur, Evidence-based health policy: context and utilisation. *Social Science & Medicine*, 2004. 58(1): 207-17.
4. Lomas, J., CHSRF knowledge transfer: decision support: a new approach to making the best healthcare management and policy choices. *Healthcare Quarterly*, 2007. 10(3).
5. Oxman, A.D., et al., SUPPORT tools for evidence-informed health policymaking (STP) 1: what is evidence-informed policymaking? *Health Research Policy and Systems*, 2009. 7(1): S1.
6. Boaz, A., S. Fitzpatrick, and B. Shaw, Assessing the impact of research on policy: a literature review. *Science and Public Policy*, 2009. 36(4): 255-270.
7. Michie, S., et al., Making psychological theory useful for implementing evidence based practice: a consensus approach. *Quality and Safety in Health Care*, 2005. 14(1): 26-33.
8. Innvær, S., et al., Health policy-makers' perceptions of their use of evidence: a systematic review. *Journal of Health Services Research & Policy*, 2002. 7(4): 239-244.
9. Brownson, R.C., et al., Communicating evidence-based information on cancer prevention to state-level policy makers. *Journal of the National Cancer Institute*, 2011. 103(4): 306-316.
10. Bogenschneider, K. and T.J. Corbett, *Evidence-based policymaking: insights from policy-minded researchers and research-minded policymakers*. 2011: Routledge.
11. Sorian, R. and T. Baugh, Power of information: closing the gap between research and policy. *Health Affairs*, 2002. 21(2): 264-273.
12. Fielding, J.E. and T.R. Frieden, Local knowledge to enable local action. *American Journal of Preventive Medicine*, 2004. 27(2): 183-184.
13. Kreuter, M.W., et al., Narrative communication in cancer prevention and control: a framework to guide research and application. *Annals of Behavioral Medicine*, 2007. 33(3): 221-235.
14. Slater, M.D., et al., A test of conversational and testimonial messages versus didactic presentations of nutrition information. *Journal of Nutrition Education and Behavior*, 2003. 35(5): 255-259.
15. Brownson, R.C., et al., Evidence-based interventions to promote physical activity: what contributes to dissemination by state health departments. *American Journal of Preventive Medicine*, 2007. 33(1): S66-S78.
16. Campbell, D., et al., Evidence Check: knowledge brokering to commission research reviews for policy. *Evidence & Policy: A Journal of Research, Debate and Practice*, 2011. 7(1): 97-107.
17. Boaz, A., et al., A multitude of syntheses: a comparison of five approaches from diverse policy fields. *Evidence & Policy*, 2006. 2(4): 479-502.
18. Mays, N., C. Pope, and J. Popay, Systematically reviewing qualitative and quantitative evidence to inform management and policy-making in the health field. *Journal of Health Services Research & Policy*, 2005. 10(suppl 1): 6-20.
19. Dobbins, M., et al., Public health decision-makers' informational needs and preferences for receiving research evidence. *Worldviews on Evidence-Based Nursing*, 2007. 4(3): 156-163.
20. Ritter, A., How do drug policy makers access research evidence? *International Journal of Drug Policy*, 2009. 20(1): 70-75.
21. Ward, V.L., A.O. House, and S. Hamer, Knowledge brokering: exploring the process of transferring knowledge into action. *BMC Health Services Research*, 2009. 9(1): 1.
22. Dagenais, C., L. Queuille, and V. Ridde, Evaluation of a knowledge transfer strategy from a user fee exemption program for vulnerable populations in Burkina Faso. *Global Health Promotion*, 2013. 20(1 suppl): 70-79.
23. Graham, I.D., et al., Lost in knowledge translation: time for a map? *Journal of Continuing Education in the Health Professions*, 2006. 26(1): 13-24.
24. Weiss, C.H., Knowledge creep and decision accretion. *Science Communication*, 1980. 1(3): 381-404.
25. Pelz, D.C., *Some expanded perspectives on use of social science in public policy*, in *Major Social Issues: A Multidisciplinary View*, M.J. Yinger and S. Cutler, Editors. 1978, Free Press: New York. p. 346-57.
26. Greenhalgh, T., et al., Diffusion of innovations in service organizations: systematic review and recommendations. *The Milbank Quarterly*, 2004. 82(4): 581-629.

27. Dobbins, M., R. Cockerill, and J. Barnsley, Factors affecting the utilization of systematic reviews. A study of public health decision makers. *International Journal of Technology Assessment in Health Care*, 2001. 17(2): 203-14.
28. Dobbins, M., et al., Factors of the innovation, organization, environment, and individual that predict the influence five systematic reviews had on public health decisions. *International Journal of Technology Assessment in Health Care*, 2001. 17(04): 467-478.
29. Rogers, E.M., *Diffusion of innovations*. 5th ed. 2003, New York: Free Press, Simon and Schuster.
30. Haines, A. and R. Jones, Implementing findings of research. *BMJ*, 1994. 308(6942): 1488.
31. Estabrooks, C.A., Will evidence-based nursing practice make practice perfect? *Canadian Journal of Nursing Research*, 1998. 30: 15-36.
32. Meyer, A.D. and J.B. Goes, Organizational assimilation of innovations: a multilevel contextual analysis. *Academy of Management Journal*, 1988. 31(4): 897-923.
33. Pettengill, M.M., D.A. Gillies, and C.C. Clark, Factors encouraging and discouraging the use of nursing research findings. *Image: The Journal of Nursing Scholarship*, 1994. 26(2): 143-148.
34. Cockerill, R. and J. Barnsley. *Innovation theory and its applicability to our understanding of the diffusion of new management practices in health care organizations*. in *Healthcare Management Forum*. 1997. SAGE Publications.
35. Kimberly, J.R. and M.J. Evanisko, Organizational innovation: the influence of individual, organizational, and contextual factors on hospital adoption of technological and administrative innovations. *Academy of Management Journal*, 1981. 24(4): 689-713.
36. Dobbins, M., et al., A randomized controlled trial evaluating the impact of knowledge translation and exchange strategies. *Implementation Science*, 2009. 4(1): 61.
37. Kreuter, M.W. and R.J. Wray, Tailored and targeted health communication: strategies for enhancing information relevance. *American Journal of Health Behavior*, 2003. 27(1): S227-S232.
38. Canadian Health Services Research Foundation, *The theory and practice of knwoeldge brokering in Canda's health system: A report based on a CHSRF national consultation and a literature review* 2003 Canadian Health Services Research Foundation: Ottawa, Canada.
39. Dwan, K.M., P. McInnes, and S. Mazumdar, Measuring the success of facilitated engagement between knowledge producers and users: a validated scale. *Evidence & Policy*, 2015. 11(2): 239-252.
40. Nutley, S.M., I. Walter, and H.T.O. Davies, *Using evidence: how research can inform public services*. 2007, Bristol: The Policy Press.
41. Cooper, A., Knowledge mobilisation in education across Canada: a cross-case analysis of 44 research brokering organisations. *Evidence & Policy*, 2014. 10(1): 29-59.
42. Lomas, J., Using 'linkage and exchange' to move research into policy at a Canadian foundation. *Health Affairs*, 2000. 19(3): 236-240.
43. Caplan, N., 2-Communities theory and knowledge utilization. *American Behavioral Scientist*, 1979. 22(3): 459-470.
44. Contandriopoulos, D., et al., Knowledge exchange processes in organizations and policy arenas: a narrative systematic review of the literature. *The Milbank Quarterly*, 2010. 88(4): 444-483.
45. Kothari, A., et al., Indicators at the interface: managing policymaker-researcher collaboration. *Knowledge Management Research & Practice*, 2011. 9(3): 203-214.
46. Shaxson, L., et al., *Expanding our understanding of K* (Kt, KE, Ktt, KMb, KB, KM, etc.): a concept paper emerging from the K* conference held in Hamilton, Ontario, Canada, April 2012*. 2012, United Nations University.
47. Hawkes, S., et al., Strengthening capacity to apply health research evidence in policy making: experience from four countries. *Health policy and planning*, 2016. 31(2): 161-170.
48. Buse, K., N. Mays, and G. Walt, *Making health policy*. 2012: McGraw-Hill Education (UK).
49. Weiss, C.H., The many meanings of research utilization. *Public Administration Review*, 1979. 39(5): 426-431.
50. Lavis, J.N., et al., SUPPORT tools for evidence-informed health policymaking (STP) 9: assessing the applicability of the findings of a systematic review. *Health Research Policy and Systems*, 2009. 7(1): S9.
51. Block, M.A.G. and A. Mills, Assessing capacity for health policy and systems research in low and middle income countries. *Health Research Policy and Systems*, 2003. 1(1): 1.
52. Department for International Development. *Capacity building in research*. Department for International Development practice papers 2010 (cited 3 Jun 2017); Available from: https://www.gov.uk/government/uploads/system/uploads/attachment_data/file/187568/HTN_Capacity_Building_Final_21_06_10.pdf.

53. Ward, V., A. House, and S. Hamer, Developing a framework for transferring knowledge into action: a thematic analysis of the literature. *Journal of Health Services Research & Policy*, 2009. 14(3): 156-164.
54. Moore, G., et al., What works to increase the use of research in population health policy and programmes: a review. *Evid Policy*, 2011. 7.
55. Hoeijmakers, M., J. Harting, and M. Jansen, Academic Collaborative Centre Limburg: a platform for knowledge transfer and exchange in public health policy, research and practice? *Health policy*, 2013. 111(2): 175-183.
56. Guston, D.H., Boundary organizations in environmental policy and science: an introduction. *Science, Technology, & Human Values*, 2001. 26(4): 399-408.
57. Bartunek, J., et al., Sharing and expanding academic and practitioner knowledge in health care. *Journal of Health Services Research & Policy*, 2003. 8 Suppl 2: 62-8.
58. Jansen, M.W. and M. Hoeijmakers, A masterclass to teach public health professionals to conduct practice-based research to promote evidence-based practice: a case study from The Netherlands. *Journal of Public Health Management and Practice*, 2013. 19(1): 83-92.
59. Jansen, M.W., et al., Working at the nexus between public health policy, practice and research. Dynamics of knowledge sharing in the Netherlands. *Health Research Policy and Systems*, 2012. 10(1): 1.
60. Kothari, A., S. Birch, and C. Charles, "Interaction" and research utilisation in health policies and programs: does it work? *Health Policy*, 2005. 71(1): 117-25.
61. Rich, R.F., Measuring knowledge utilization: processes and outcomes. *Knowledge and Policy*, 1997. 10(3): 11-24.
62. Kothari, A., S.L. Sibbald, and C.N. Wathen, Evaluation of partnerships in a transnational family violence prevention network using an integrated knowledge translation and exchange model: a mixed methods study. *Health Research Policy and Systems*, 2014. 12(1): 1.
63. Mitchell, P., et al., Partnerships for knowledge exchange in health services research, policy and practice. *Journal of Health Services Research & Policy*, 2009. 14(2): 104-111.
64. Wathen, C.N., et al., Talk, trust and time: a longitudinal study evaluating knowledge translation and exchange processes for research on violence against women. *Implementation science*, 2011. 6(1): 1.
65. Goldman, K.D. and K.J. Schmalz, Being well-connected: starting and maintaining successful partnerships. *Health Promotion Practice*, 2008. 9(1): 5-8.
66. Provan, K.G. and P. Kenis, Modes of network governance: structure, management, and effectiveness. *Journal of Public Administration Research and Theory*, 2008. 18(2): 229-252.
67. Smith, K., et al., Partners in health? A systematic review of the impact of organizational partnerships on public health outcomes in England between 1997 and 2008. *Journal of Public Health*, 2009. 31(2): 210-221.
68. Hayes, S.L., et al., Collaboration between local health and local government agencies for health improvement. *The Cochrane Library*, 2012.
69. Thomson, A.M. and J.L. Perry, Collaboration processes: inside the black box. *Public Administration Review*, 2006. 66(s1): 20-32.
70. Braun, V. and V. Clarke, Using thematic analysis in psychology. *Qualitative Research in Psychology*, 2006. 3(2): 77-101.
71. Langlois, E.V., et al., Enhancing evidence informed policymaking in complex health systems: lessons from multi-site collaborative approaches. *Health Research Policy and Systems*, 2016. 14(1): 20.
72. Naude, C.E., et al., Research evidence and policy: qualitative study in selected provinces in South Africa and Cameroon. *Implementation Science*, 2015. 10(1): 126.
73. World Health Organization, Changing mindsets: strategy on health policy and systems research. *Geneva: WHO*, 2012.
74. Aarons, G.A., M. Hurlburt, and S.M. Horwitz, Advancing a conceptual model of evidence-based practice implementation in public service sectors. *Administration and Policy in Mental Health and Mental Health Services Research*, 2011. 38(1): 4-23.
75. Oliver, K., et al., A systematic review of barriers to and facilitators of the use of evidence by policymakers. *BMC Health Services Research*, 2014. 14(1): 2.
76. Walt, G. and L. Gilson, Reforming the health sector in developing countries: the central role of policy analysis. *Health Policy Plan*, 1994. 9(4): 353-70.
77. Pappaioanou, M., et al., Strengthening capacity in developing countries for evidence-based public health:: the data for decision-making project. *Social Science & Medicine*, 2003. 57(10): 1925-1937.
78. Davis, P., *Problems, politics, and processes: public health sciences and policy in developed countries*, in *Textbook of Public Health*, R. Detels, et al., Editors. 2002, Oxford University Press: New York. p. 937-950.

79. Frank, J.W., Public health policy and the quality of epidemiological evidence: how good is good enough? *Journal of Public Health Policy*, 1985. 6(3): 313-321.
80. Goodman, R.A., P.L. Remington, and R.J. Howard, Communicating information for action. *Morbidity and Mortality Weekly Report*, 1992. 41 Suppl: 143-7.
81. Frerichs, R.R., Epidemiologic surveillance in developing countries. *Annual Review of Public Health*, 1991. 12(1): 257-280.
82. Peirson, L., et al., Building capacity for evidence informed decision making in public health: a case study of organizational change. *BMC Public Health*, 2012. 12(1): 137.
83. Canadian Health Services Research Foundation (CHSRF) and Ontario Home Care Research Network, Bringing knowledge home: embedding evidence in decision-making *Promising Practices in Research Use*, 2006. 5.
84. Kotter, J.P., *Leading change*. 1996: Harvard Business Press.
85. Senge, P.M., *The fifth discipline: the art and practice of the learning organization*. 2006: Broadway Business.
86. Rycroft-Malone, J., The PARIHS framework—a framework for guiding the implementation of evidence-based practice. *Journal of Nursing Care Quality*, 2004. 19(4): 297-304.
87. Rolle, I.V., et al., Leadership in strategic information (LSI) building skilled public health capacity in Ethiopia. *BMC Research Notes*, 2011. 4(1): 292.
88. Thacker, S.B., A.L. Dannenberg, and D.H. Hamilton, Epidemic intelligence service of the Centers for Disease Control and Prevention: 50 years of training and service in applied epidemiology. *American Journal of Epidemiology*, 2001. 154(11): 985-92.
89. Farel, A., K. Umble, and B. Polhamus, Impact of an online analytic skills course. *Evaluation & the Health Professions*, 2001. 24(4): 446-459.
90. Shroff, Z., et al., Incorporating research evidence into decision-making processes: researcher and decision-maker perceptions from five low- and middle-income countries. *Health Research Policy and Systems*, 2015. 13: 70.
91. Lavis, J.N., et al., Assessing country-level efforts to link research to action. *Bulletin of the World Health Organization*, 2006. 84(8): 620-628.
92. Hyder, A.A., et al., National policy-makers speak out: are researchers giving them what they need? *Health Policy and Planning*, 2010: czq020.
93. El-Jardali, F., et al., Capturing lessons learned from evidence-to-policy initiatives through structured reflection. *Health Research Policy and Systems*, 2014. 12(1): 1.
94. American University of Beirut. *Knowledge to Policy. K2P Policy Briefs*. (cited 13 Dec 2016); Available from: www.aub.edu.lb/k2p/products/Pages/k2pbriefs.aspx.
95. Cheung, A., et al., Climate for evidence-informed health systems: A print media analysis in 44 low-and middle-income countries that host knowledge-translation platforms. *Health Research Policy and Systems*, 2011. 9(1): 1.
96. Jacobson, N., D. Butterill, and P. Goering, Development of a framework for knowledge translation: understanding user context. *Journal of Health Services Research & Policy*, 2003. 8(2): 94-99.
97. Traynor, R., K. DeCorby, and M. Dobbins, Knowledge brokering in public health: a tale of two studies. *Public Health*, 2014. 128(6): 533-544.
98. Thompson, G.N., C.A. Estabrooks, and L.F. Degner, Clarifying the concepts in knowledge transfer: a literature review. *Journal of Advanced Nursing*, 2006. 53(6): 691-701.
99. Hargadon, A.B., Brokering knowledge: linking learning and innovation. *Research in Organizational Behavior*, 2002. 24: 41-85.
100. McCormack, B., et al., A realist review of interventions and strategies to promote evidence-informed healthcare: a focus on change agency. *Implementation Science*, 2013. 8(1): 107.
101. Yin, R.K., *Case study research: design and methods*. 4th ed. 2009, California: SAGE Publications.
102. Miles, M.B. and A.M. Huberman, *Qualitative data analysis: an expanded sourcebook*. 2nd ed. 1994, Thousands Oaks, CA: SAGE.
103. Patton, M.Q., *Qualitative research and evaluation methods*. 3rd ed. 2002, Thousand Oaks, CA: SAGE.
104. Uneke, C.J., et al., Promotion of evidence-informed health policymaking in Nigeria: bridging the gap between researchers and policymakers. *Global public health*, 2012. 7(7): 750-765.
105. Livny, E., A. Mehendale, and A. Vanags, Bridging the research policy gaps in developing and transition countries: Analytical lessons and proposals for action. *A synthesis of findings from the Global Development Network's Bridging Research and Policy Project, 8 December 2006*, 2006.
106. Lavis, J.N., et al., SUPPORT tools for evidence-informed health policymaking (STP) 3: setting priorities for supporting evidence-informed policymaking. *Health Research Policy and Systems*, 2009. 7(1): S3.

107. Jönsson, K., et al., Health systems research in Lao PDR: capacity development for getting research into policy and practice. *Health Research Policy and Systems*, 2007. 5(1): 1.
108. Poulos, R.G., A.B. Zwi, and S.R. Lord, Towards enhancing national capacity for evidence informed policy and practice in falls management: a role for a" Translation Task Group"? *Australia and New Zealand Health Policy*, 2007. 4(1): 1.
109. Johnson, N. and J. Lavis, Procedures Manual for the "Evaluating knowledge-translation platforms in low- and middle-income countries" study. *Hamilton, Canada: McMaster University Program in Policy Decision-Making*, 2009. 17.
110. Giorgi, A., *Sketch of a psychological phenomenological method*, in *Phenomenology and psychological research: essays*, A. Giorgi, Editor. 1985, Duquesne University: Pittsburgh, PA.
111. Uneke, C.J., et al., Enhancing the capacity of policy-makers to develop evidence-informed policy brief on infectious diseases of poverty in Nigeria. *International Journal of Health Policy and Management*, 2015a. 4(9): 599-610.
112. Green, A. and S. Bennett, *Sound choices: enhancing capacity for evidence-informed health policy* 2007, World Health Organization: Geneva. Available from: http://www.who.int/alliance-hpsr/resources/Alliance_BR.pdf.
113. Bennett, S., et al. *What must be done to enhance capacity for health systems research?* in *Background paper for the Global Symposium on Health Systems Research*. 2010. Montreux, Switzerland, 16-19 Nov.
114. Canadian Coalition for Global Health Research. *Why mentorship? (Module three)*. Mentorship and Leadership Resources: Mentoring Modules 2007 (cited 2 Apr 2017); Available from: http://www.ccghr.ca/wp-content/uploads/2013/05/Mentoring_Module3_Why-Mentorship_e.pdf.
115. Uneke, C.J., et al., Implementation of a health policy advisory committee as a knowledge translation platform: the Nigeria experience. *Int J Health Policy Manag*, 2015b. 4(3): 161–168.
116. Haines, A., S. Kuruvilla, and M. Borchert, Bridging the implementation gap between knowledge and action for health. *Bulletin of the World Health Organization*, 2004. 82(10): 724-731.
117. Choi, B.C.K., et al., Can scientists and policy makers work together? *Journal of Epidemiology and Community Health*, 2005. 59(8): 632.
118. Baxter, P. and S. Jack, Qualitative case study methodology: Study design and implementation for novice researchers. *The Qualitative Report*, 2008. 13(4): 544-559.
119. Uneke, C.J., et al., Policy brief: improving maternal and child healthcare programme using community-participatory interventions in Ebonyi State Nigeria. *International Journal of Health Policy and Management*, 2014. 3(5): 283–287.
120. Waqa, G., et al., Knowledge brokering between researchers and policymakers in Fiji to develop policies to reduce obesity: a process evaluation. *Implementation Science*, 2013. 8(1): 74.
121. Mavoa, H., et al., Knowledge exchange in the Pacific: the TROPIC (Translational Research into Obesity prevention Policies for Communities) project. *BMC public health*, 2012. 12(1): 1.
122. Lavis, J.N., et al., SUPPORT tools for evidence-informed health policymaking (STP) 5: using research evidence to frame options to address a problem. *Health Research Policy and Systems*, 2009. 7(1): 1.
123. Oxman, A.D., et al., SUPPORT tools for evidence-informed health policymaking (STP) 2: improving how your organisation supports the use of research evidence to inform policymaking. *Health Research Policy and Systems*, 2009. 7(1): 1.
124. Waters, E., et al., An exploratory cluster randomised controlled trial of knowledge translation strategies to support evidence-informed decision-making in local governments (The KT4LG study). *BMC Public Health*, 2011. 11(1): 34.
125. van der Heide, I., et al., Implementation of a tool to enhance evidence-informed decision making in public health: identifying barriers and facilitating factors. *Evidence & Policy*, 2016. 12(2): 183-197.
126. Nutley, S., I. Walter, and H.T. Davies, From knowing to doing a framework for understanding the evidence-into-practice agenda. *Evaluation*, 2003. 9(2): 125-148.
127. Hanney, S., et al., The utilisation of health research in policy-making: concepts, examples and methods of assessment. *Health Research Policy and Systems*, 2003. 13(1).
128. Young, K., et al., Social science and the evidence-based policy movement. *Social Policy and Society*, 2002. 1(03): 215-224.
